# Structural determinants of quadriceps atrophy following ACL injury: Evidence for fibre atrophy without fibre loss or overt peripheral denervation

**DOI:** 10.1113/JP291436

**Published:** 2026-08-23

**Authors:** Luke Stoneback, Brianna Buchanan, Hadi W. Hussein, Dania Ali, Sairub Naaz, Keegan Gustafson, Leah Vaikutis, Lucas Damouni, Peter C. D. Macpherson, Lindsey K. Lepley

**Affiliations:** ^1^ School of Kinesiology University of Michigan Ann Arbor Michigan USA; ^2^ Michigan State University College of Osteopathic Medicine East Lansing Michigan USA; ^3^ Saint Louis University School of Medicine St. Louis Missouri USA; ^4^ Department of Molecular and Integrative Physiology University of Michigan Ann Arbor Michigan USA

**Keywords:** anterior cruciate ligament, muscle atrophy, rehabilitation

## Abstract

**Abstract:**

Quadriceps atrophy is a well‐established consequence of anterior cruciate ligament (ACL) injury, yet the mechanisms driving this process remain poorly characterized. Specifically, it is unclear whether atrophy reflects decreases in fibre cross‐sectional area (CSA) or fibre number, and whether the neural inhibition accompanying ACL injury extends to peripheral neuromuscular junctions (NMJs). Using a rat model of non‐invasive ACL injury and whole‐muscle histological analysis, we quantified changes in vastus lateralis fibre CSA, fibre number, fibre‐type distribution, and NMJ integrity and function at early post‐injury timepoints. By 7 days post‐injury, fibre CSA was significantly reduced in both sexes across all Type II subtypes, with deficits persisting at 14 days. Total fibre number was maintained, demonstrating that whole‐muscle atrophy is driven primarily by reductions in fibre CSA rather than fibre loss. At 3 days post‐injury, no significant difference in NMJ innervation and compound muscle action potential amplitude were observed, suggesting that early atrophy is not preceded by peripheral denervation. Reductions in electromyographic amplitude and stance duration during treadmill walking indicated decreased muscle activation and limb loading following injury. Shifts in fibre‐type distribution to IIb were also observed. This work clarifies the structural basis of early quadriceps atrophy following ACL injury, demonstrating that quadriceps atrophy is likely not preceded by peripheral denervation and is characterized primarily by reductions in fibre CSA. These findings support rehabilitation strategies that target fibre growth, but the persistent nature of quadriceps atrophy in patients suggests that current approaches may be insufficient and require further optimization.

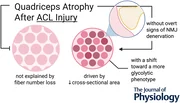

**Key points:**

- Anterior cruciate ligament (ACL) injury results in persistent quadriceps atrophy, yet whether deficits in whole‐muscle size result from reductions in fibre cross‐sectional area or number, and whether the integrity of neuromuscular junctions is impaired, remains unclear.
- Using a rat model of ACL injury and whole‐muscle histology, we demonstrate that quadriceps atrophy is driven by decreased fibre cross‐sectional area, not fibre loss, and that this affects all Type II subtypes.
- Quadriceps neuromuscular junctions remained structurally and functionally intact, demonstrating that early quadriceps atrophy is not preceded by denervation.
- Shifts in fibre‐type composition toward a more glycolytic phenotype were observed, consistent with patterns reported in models of muscle unloading and disuse.
- By identifying fibre‐level atrophy without fibre loss, these findings highlight a key therapeutic target and support strategies that target fibre growth to address the persistent atrophy that plagues ACL‐injured patients.

## Graphical abstract

**Abstract figure legend** Quadriceps atrophy after ACL injury is characterized by reduced muscle fibre cross‐sectional area without evidence of fibre loss or overt denervation. Reductions in whole‐muscle size are explained by decreases in muscle fibre cross‐sectional area, while neuromuscular junctions (NMJs) remain structurally and functionally intact. ACL injury is also associated with a shift toward a more glycolytic muscle phenotype.

## Introduction

Anterior cruciate ligament (ACL) injury is a common and costly injury that results in well‐documented complications that extend beyond the knee joint itself (Ajuied et al., 2014; Birchmeier et al., 2020; Mall et al., 2014; Mather et al., 2013). Among the most evident of these is persistent quadriceps atrophy, with studies consistently showing that these reductions in muscle size persist for months to years despite intensive rehabilitation (Birchmeier et al., 2020; Ito et al., 2025; Thomas et al., 2016). This has motivated work aimed at uncovering the aetiology of quadriceps atrophy in the hopes that gaining a better understanding of the progression and nature of this process will lead to more effective therapies to reverse it.

Our understanding of quadriceps atrophy after ACL injury has largely been shaped by observations made at the whole‐muscle level, most commonly using magnetic‐resonance imaging to quantify whole‐muscle volume and ultrasound to assess muscle thickness. While these measurements have been used to reliably measure quadriceps atrophy after injury (Losciale et al., 2025; Macleod et al., 2014; Williams et al., 2005) and reconstruction (Birchmeier et al., 2020; Hunnicutt et al., 2020; Kuenze et al., 2022; Lepley et al., 2019; Norte et al., 2018), they are limited in their ability to determine the underlying factors contributing to whole‐muscle atrophy. As demonstrated in denervation‐induced and age‐related muscle atrophy (Carlson, 2014; Horwath et al., 2025; Jackson, 2025; Lexell et al., 1983; Wilkinson et al., 2018), reductions in whole muscle size may reflect two primary mechanisms: (1) decreases in fibre cross‐sectional area (CSA) and/or (2) loss of muscle fibres. Distinguishing between these mechanisms is essential, as reductions in fibre CSA typically reflect the activation of canonical atrophy pathways (Sartori et al., 2021) in conjunction with depressed protein synthesis (Bodine, 2013), whereas fibre loss most commonly originates from denervation and impaired reinnervation (Jackson, 2025).

Of these two contributors, changes in fibre CSA have been more thoroughly investigated after ACL injury. Biopsy studies from ACL‐injured patients consistently report reductions in fibre CSA in the involved limb, providing cellular‐level insight into quadriceps atrophy (Graham et al., 2024; Noehren et al., 2016; Owen et al., 2024; Tourville et al., 2022). Whether atrophy occurs uniformly across fibre types or preferentially affects specific populations, however, remains a topic of debate. Some studies show non‐specific reductions in fibre types (Parstorfer et al., 2021; Toth et al., 2020), whereas others describe selective atrophy of Type II fibres (Latham et al., 2024; Leszczynski et al., 2021; Noehren et al., 2016). Sex‐based differences have also been reported with female patients exhibiting greater vastus lateralis (VL) CSA deficits (Owen et al., 2024) and divergent transcriptomic profiles (Keeble, Owen et al., 2026) compared with males, in contrast to whole‐muscle imaging data showing similar size deficits across sexes (Lindström et al., 2013; Strandberg et al., 2013). In addition to changes in fibre size, studies have also investigated changes in fibre‐type distribution following ACL injury and reconstruction with mixed findings across timepoints (Flück et al., 2018; Graham et al., 2024; Noehren et al., 2016). Taken together, these data demonstrate consistent reductions in fibre CSA following ACL injury, yet important questions remain regarding fibre‐type specificity, sex‐based differences and phenotypic transitions, limitations that likely reflect the constrained sampling inherent to biopsy‐based approaches.

In contrast to changes in fibre CSA, far less is known about the extent to which changes in fibre number contribute to quadriceps atrophy after ACL injury. Reductions in fibre number are primarily found in atrophic conditions that involve fibre denervation (Ham & Rüegg, 2018; Kostrominova, 2022). Although denervation has not been directly demonstrated after ACL injury, it is well‐documented that quadriceps atrophy occurs in the context of a complex series of neurological events that inhibit quadriceps activation (McPherson et al., 2023; Pietrosimone et al., 2022). To date, the extent to which these neural changes involve the quadriceps neuromuscular junctions (NMJs) remains unclear. However, elevated markers of neuromuscular distress, specifically neuronal cell adhesion molecule (NCAM), have been identified in both ACL‐injured patients and preclinical models of ACL injury (Fry et al., 2017; Hunt et al., 2022), alongside elevated expression of denervation‐responsive genes (cholinergic receptor nicotinic beta 1 subunit (CHRNB1)) after ACL reconstruction (Keeble, Gonzalez et al., 2026). In other neuromuscular and disuse‐related conditions, increased NCAM and CHRNB1 expression is often associated with NMJ instability, denervation and impaired reinnervation (Chipman et al., 2014; Covault & Sanes, 1985; Keeble, Gonzalez et al., 2026; Rafuse et al., 2000; Sirago et al., 2023). Given that these neurological changes coincide with quadriceps atrophy and weakness, it is formally possible that higher‐order inhibition after ACL injury may result in downstream NMJ denervation and thus necessitates careful investigation.

Clarifying whether persistent quadriceps atrophy after ACL injury is a consequence of reduced fibre size, fewer fibres, or both, is fundamental to understanding the mechanisms underlying whole‐muscle loss and informing targeted treatment strategies. Additionally, determining whether NMJ integrity and function are preserved during the early post‐injury period is essential for distinguishing the possible role of peripheral denervation in the development of quadriceps atrophy. Utilizing a preclinical model that enables whole‐muscle analysis, the purpose of this study was to evaluate changes in fibre CSA, fibre number, fibre‐type distribution and NMJ integrity and function in the VL following ACL injury.

## Methods

### Ethical approval

A total of 88 Long Evans rats (16 weeks of age) were obtained (Envigo Laboratories Indianapolis, IN, United States) under the approval of the Institutional Animal Care and Use Committee (IACUC approval #PRO00012449) at the University of Michigan. Animals were maintained in accordance with the National Institutes of Health guidelines on the care and use of laboratory animals. All rats were housed in standard sized cages on a 12:12 light–dark cycle and allowed access to both food and water *ad libitum* for the duration of the study. The investigators understand the ethical principles under which *The Journal of Physiology* operates and confirm that this work complies with the journal's animal ethics checklist, as laid out by Grundy and O'Halloran (Grundy, 2015; O'Halloran, 2024).

### Animals and experimental groups

All fibre morphology, fibre type, EMG and NMJ analyses were conducted in the VL. The VL was selected to facilitate comparison with prior preclinical work in this ACL injury model (Davi et al., 2022; Hunt et al., 2022; Noh et al., 2024) and to enhance translational relevance to human biopsy‐based studies, where the VL is the most commonly sampled quadriceps muscle.

For fibre morphology and fibre‐type analyses, male and female rats were assigned to the following groups (*n* = 6 m, *n* = 6 F per group): uninjured control (T0), 7‐day ACL‐injured (T7) and 14‐day ACL‐injured (T14). The 7‐ and 14‐day timepoints were chosen because these intervals capture the development of quadriceps atrophy in our non‐invasive ACL injury model, aligning with our prior and other preclinical studies demonstrating significant decreases in VL size during this period (Davi et al., 2022; Hunt et al., 2022; Kaneguchi et al., 2023, 2025; Latham et al., 2024).

To get a proxy of muscle activation after injury, an additional cohort of rats (*n* = 8 M, *n* = 8 F) was instrumented with electromyography (EMG) electrodes on the right (affected) hindlimb and stance duration and VL EMG were recorded during level treadmill walking (16 m/min) prior to and serially following ACL injury for 14 days.

For assessments of NMJ integrity, immunohistochemistry was performed on the affected (right) and contralateral (left) VLs of 3‐day ACL‐injured rats (*n* = 6 M, *n* = 6 F). For assessments of NMJ function via compound muscle action potentials (CMAPs), the affected (right) limb of a separate cohort of 3‐day ACL‐injured rats (*n* = 6 M, *n* = 6 F) was compared with the right limb of age‐matched uninjured control animals (*n* = 6 M, *n* = 6 F); bilateral CMAP recordings were not feasible in this experiment, precluding within‐subject paired comparisons. The 3‐day post‐injury timepoint was selected to capture the early transition between acute neural inhibition due to pain and effusion (Pietrosimone et al., 2022) and the onset of measurable muscle atrophy following ACL injury in this model (Davi et al., 2022). Prior work demonstrates a rapid elevation in NCAM expression within the quadriceps as early as 6 h post‐injury, indicating neuromuscular distress and potential disruption of NMJ stability (Hunt et al., 2022). However, structural atrophy is not yet fully established at this stage, whereas robust quadriceps atrophy is evident by 7 days post‐injury. Accordingly, the 3‐day timepoint represents an intermediate window to determine whether early neuromuscular alterations occur prior to changes in fibre CSA, number and fibre‐type distribution.

At study completion, all animals were killed via carbon dioxide inhalation followed by bilateral pneumothorax, in accordance with the AVMA *Guidelines for the Euthanasia of Animals*.

### Non‐invasive ACL injury

ACL injury was induced using a single load of tibial compression (White et al., 2022). The device consists of two custom‐built loading platforms: the top knee stage is rigidly mounted to a linear actuator (DC linear actuator L16‐63‐12‐P, Phidgets) that positions the right hindlimb in 30° of dorsiflexion and 100° of knee flexion while providing room for anterior subluxation of the tibia relative to the femur; the bottom stage holds the flexed knee and is mounted directly above a load cell (HDM Inc., PW6D). Rats were anaesthetized (5% induction and 1 L/min oxygen; maintained at 2% via nose cone with 500 mL/min oxygen) and the right hindlimb was subject to single load of tibial compression at a speed of 8 mm/s. ACL rupture was identified by a release of compressive force during loading, monitored via a custom program (LabVIEW, National Instruments). Post‐injury, a Lachman's test was performed to confirm an ACL rupture had occurred and ensure no contraindications were present (e.g. fractures). Post‐mortem evaluations of the knee joint provided additional confirmation of isolated ACL injury.

### Body composition

Whole‐body composition was assessed at baseline (prior to injury) and at endpoint (immediately prior to tissue harvest at each respective post‐injury timepoint) to characterize systemic changes associated with ACL injury. Body mass, lean mass and fat mass were measured using quantitative magnetic resonance (EchoMRI; EchoMRI LLC, Houston, TX, USA).

### Wet muscle weight

At study completion, under deep anaesthesia, bilateral quadriceps muscles were carefully excised for wet muscle weight (WMW) measurement from control, 7‐day ACL‐injured and 14‐day ACL‐injured cohorts. WMWs were recorded to the nearest 0.1 mg on an analytical balance. Bilateral VLs were snap‐frozen in liquid nitrogen cooled isopentane and stored at −80°C for immunofluorescence analyses.

### Fibre typing

**Figure 1:**
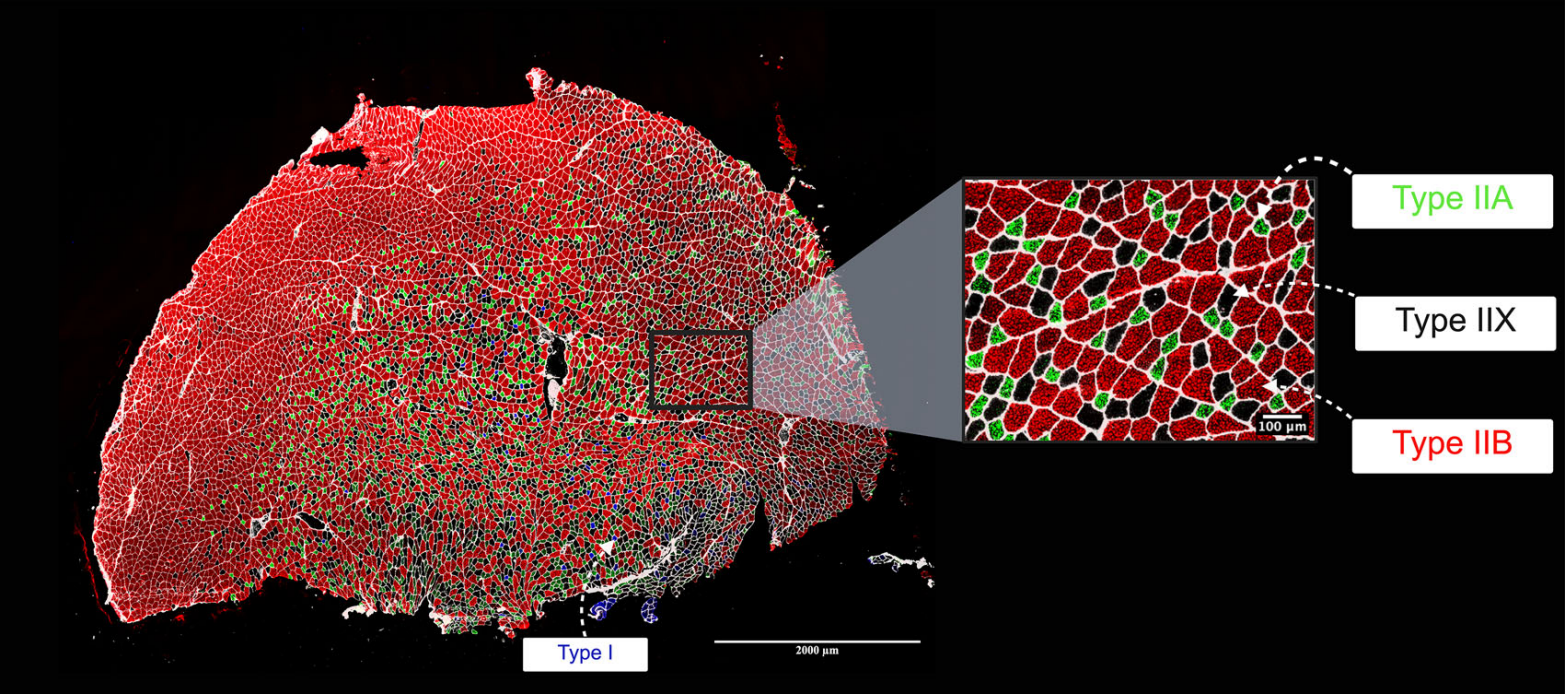
Representative whole‐muscle cross‐section of the vastus lateralis Immunofluorescence image of a transverse cryosection through the muscle belly of the vastus lateralis, acquired at 10× magnification on a Leica Stellaris 5 confocal microscope and assembled into a single composite using the automated tile‐stitching function in Leica LAS X software. Wheat germ agglutinin (WGA; white) labels fibre borders; and antibodies against myosin heavy‐chain isoforms identify Type I (blue), Type IIa (green) and Type IIb (red) fibres; Type IIx fibres appear unstained (black) and were identified by exclusion, as no IIx‐specific antibody was included in the panel. Fibre‐type identities and cross‐sectional areas were extracted from whole‐muscle sections using the Cellpose segmentation and Labels2ROIs pipeline described in the *Methods*. Scale bar = 2000 µm for tile scanned image; 100 µm for call‐out.

Transverse cryosections (6–8 µm) from the muscle belly of bilateral VLs were cut on a cryostat, mounted on glass slides, air‐dried overnight at room temperature, and stored at −20°C prior to staining. On the day of staining, sections were rehydrated in PBS (2 × 3 min) and blocked in 5% normal goat serum for 30 min at room temperature. Slides were then incubated overnight at 4°C with primary antibodies against myosin heavy chain (MyHC) isoforms to identify fibre types: Type I fibres (BA.D5, IgG2b, 1:100; DSHB), Type IIa fibres (SC.71, IgG1, 1:100; DSHB) and Type IIb fibres (BF.F3, IgM, 1:50; DSHB). Type IIx fibres were unstained. The following day, sections were washed in PBS (3 × 5 min) and incubated for 60 min at room temperature with secondary antibodies: goat anti‐mouse IgG2b Alexa Fluor 647 (1:250; Invitrogen # A‐21242) for Type I, goat anti‐mouse IgG1 Alexa Fluor 488 (1:500; Invitrogen #A‐21121) for Type IIa, and goat anti‐mouse IgM Alexa Fluor 555 (1:250; Invitrogen #A‐21426) for Type IIb fibres. Sections were co‐stained with wheat germ agglutinin (WGA) conjugated to Alexa Fluor 405 (WGA‐405; 1:200–1:400; Invitrogen #W11261) to outline the sarcolemma. Slides were then washed in PBS, mounted with mounting medium (Fluorescence Mounting Medium; Agilent #S302380‐2) and stored protected from light at −20°C until imaging. Images were acquired using a scanning confocal microscope equipped with a white light laser (Leica Stellaris 5; Leica Microsystems, Wetzlar, Germany) and tuned to 405, 488, 555 and 647 nm lines. Images were captured at 10× magnification using sequential line excitation and emission collection to minimize bleed‐through. A representative whole‐muscle cross‐section is presented in Fig. 1.

### Fibre analysis

To assess changes in fibre size and number in whole‐muscle VL sections, a validated high throughput fibre segmentation (Cellpose) and feature extraction (Labels2ROIs) pipeline was used (Stringer & Pachitariu, 2025; Waisman et al., 2021).

#### Segmentation

Raw .tif confocal image files were imported into ImageJ/Fiji for channel separation and composite generation. The 405 nm channel, corresponding to WGA‐labelled fibre borders, was exported as an individual grayscale .tif image for segmentation, while the remaining channels (488, 555 and 647 nm; corresponding to Type IIa, IIb and I fibres, respectively) were merged into a composite .tif image for downstream region of interest (ROI) generation. The WGA .tif images were segmented using Cellpose v3.0.1 with the cyto2 model, using automated diameter estimation and default parameters (flow threshold = 0.4; cell probability threshold = 0.0; channel configuration = 0,0). Segmentation produced .png mask files, which were overlaid with the original WGA images to visually confirm accurate boundary detection. Segmentation results, including ROI coordinates, were exported and prepared for downstream processing in the Labels2ROIs and fibre‐typing pipeline.

#### Labels2ROIs and feature extraction

Following Cellpose segmentation, the binary mask for each section was overlaid onto the fibre‐type composite images using the Labels2ROIs Fiji macro (Stringer & Pachitariu, 2025; Stringer et al., 2021; Waisman et al., 2021). This process generated composite erosion files for every muscle section, containing a comprehensive set of geometric and intensity‐based measurements for each individual fibre/ROI (Waisman et al., 2021). These files served as the raw input for analysis.

#### Fibre‐type classification

A rule‐based classification pipeline was used to assign fibre types (Type I, Type IIa and Type IIb) based on the mean grayscale intensity of each fibre ROI across the three immunolabelled channels. This approach takes advantage of the distinct intensity profiles produced by each fibre type and its respective antibody, allowing fibres to be classified by their fluorescent intensity. Following ROI extraction and feature generation with Labels2ROIs, grayscale intensity values for each fibre were imported into a Python‐based pipeline for rule‐based classification and downstream analyses. To remove outlier structures, fibres with CSA or circularity values falling outside ±2 standard deviations of the mean were removed, with absolute lower limits of 800 µm^2^ and 0.35 applied to CSA and circularity, respectively, to prevent over‐exclusion as these structures represented non‐biological artefacts (e.g. freeze artefact, merged ROIs or irregular/oblique shapes). To assess whether exclusion rates were influenced by experimental condition, the proportion of excluded fibres was compared across groups and timepoints. No systematic difference in exclusion frequency was observed between ACL‐injured and contralateral limbs, indicating that shape‐based exclusions did not disproportionately affect either condition. An 8‐bit grayscale minimum intensity threshold of 24 (experimentally determined from historic fibre‐typing images) was applied as a channel‐level threshold to support identification of Type IIx fibres, which lack antibody staining and therefore are underrepresented if background fluorescence is not accounted for in all three channels.

Fibre types were subsequently assigned according to a dominance‐based rule set. Fibres in which all three channel intensities fell below the IIx cutoff (24 grayscale units) were classified as Type IIx, consistent with the absence of antibody staining for this isoform. For fibres fluorescing above this threshold in one or more MyHC channels, the dominant fibre type was assigned when the intensity of one channel exceeded the next highest channel by >1.2‐fold and by at least 20 grayscale values. Fibres that did not meet these dominance criteria were classified as ‘ambiguous’ and conservatively excluded from CSA and fibre‐type related analyses, as their fluorescence intensity profiles did not permit confident fibre‐type resolution. Hybrid fibre identification was not attempted for similar reasons: the absence of a direct Type IIx antibody means that IIa/IIx and IIx/IIb hybrids cannot be reliably distinguished from IIx fibres or low‐intensity background staining, and potential IIa/IIb hybrids would be expected to pass through a region of low IIx‐channel signal, rendering them indistinguishable from the ambiguous class. Additionally, the rarity of Type I fibres (∼1%) additionally makes meaningful resolution of Type I/IIa hybrid populations unlikely. Across the full dataset (*n* = 617,353 fibres), ambiguous fibres accounted for 13,173 fibres (∼2.1%), confirming that the dominance‐based classification successfully resolved the vast majority (∼97.9%) of fibres with confidence, and that the proportion excluded was too small to influence the fibre type‐specific analyses reported below.

#### Fibre‐type classification validation

**Figure A1:**
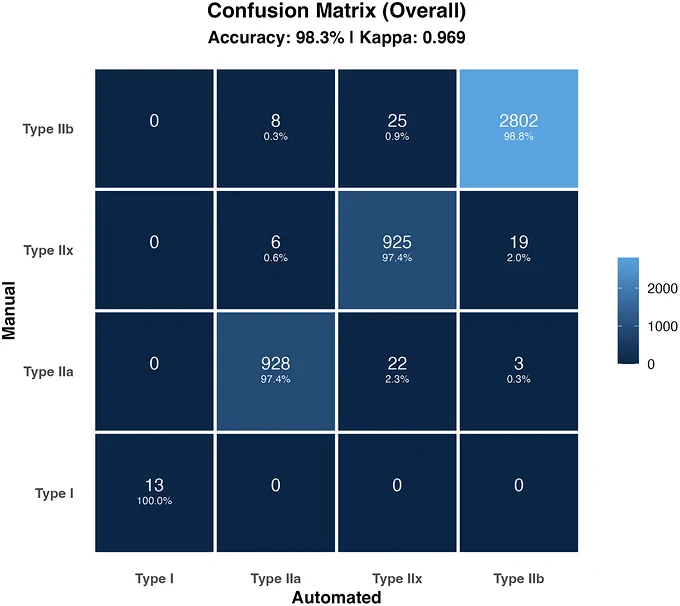
Confusion matrix for automated muscle fibre‐type classification Agreement between automated fibre‐type predictions and manual labels was evaluated using a confusion matrix across 4751 manually annotated fibres. Each cell represents the number of fibres assigned to a given manual–automated class combination. Overall classification accuracy was 98.3% with excellent agreement beyond chance (Cohen's κ = 0.969).

To validate the classification approach, automated fibre‐type assignments were compared with manual fibre identification. Ten 10× images were collected from VL muscle sections stained and imaged as described above. Fibres were then identified as Type I, IIa, IIx and IIb by a trained observer (L.D.). Automated classifications were then compared with manually assigned labels using a confusion matrix, and overall accuracy, precision, recall and Cohen's kappa were calculated to assess agreement. Validation results are presented in Fig. A1 and Table A2.

### VL electromyography instrumentation

Prior to ACL injury, an additional cohort of rats (*n* = 8 M, *n* = 8 F) were instrumented with a biocompatible, indwelling epimysial EMG electrode on the right (affected) VL (Stoneback et al., 2024) to assess muscle activity following non‐invasive ACL injury. Anaesthesia was induced using an induction chamber with 5% isoflurane and 1 L/min oxygen and maintained via a nose cone with 2% isoflurane and 500 mL/min oxygen while the rat was positioned in a stereotactic frame.

After reaching the surgical plane of anaesthesia, a 1 cm incision was made in line with the sagittal suture of the skull, and the top of the skull was exposed and cleaned. A single hole was drilled into the outer layer of the skull for the attachment of a self‐tapping ground screw (19010‐00, Fine Science Tools). An electrode interface board (EIB‐16‐QC, Neuralynx Inc.) was then secured to the skull using dental cement (Excel Formula Pourable Dental Material, St. George Technology Inc.) and bonded to the single self‐tapping ground screw. Following the attachment of the electrode interface board, an incision was made on the anterolateral portion of the right hindlimb, and blunt dissection was used to identify the VL muscle. The EMG electrode leads were then routed from the skull to the VL through a subcutaneous tunnel along the spine. At the VL, the EMG electrode was then implanted in line with the muscle fibres using 4‐0 non‐absorbable monofilament sutures. The electrode leads were pinned to the EIB and transmitted to a high‐density animal EMG system (DigitalLynx 4sX Base, Neuralynx Inc.) via a magnetic tethered cable system (HS‐16‐QC, Neuralynx Inc.). Incisions were closed using wound clips, and the rat was allowed to recover on a heated pad. Meloxicam was used to control pain for the first 72 h (three total doses (dose one administered pre‐op), 2 mg/kg subcutaneous injection).

### Electromyography and gait acquisition

Following a 10‐day recovery period, volitional VL EMG activity and stance duration were assessed during a treadmill challenge, consisting of level‐ground treadmill walking (16 m/min; Exer 3/6, Columbus Instruments). Baseline EMG and stance duration data were collected prior to ACL injury. Post‐injury data were then collected at 6 h, 3, 7 and 14 days following ACL injury in alignment with our atrophy timeline.

VL EMG activity was recorded at 2000 Hz using the DigitalLynx 4sX Base system (Neuralynx Inc.) and synchronized with motion capture data acquired at 200 frames per second using OptiTrack Prime Colour cameras (Motive software, v2.1.1). Analogue signals were routed through a Neuralynx MDR50 Breakout Board to a Krohn‐Hite 3382 Dual Channel Filter (high pass 75 Hz; low pass 1000 Hz; gain of 10), then interfaced with a data acquisition system (National Instruments USB‐6212) and OptiTrack Esync 2 device for synchronization.

### Electromyography and gait data processing

EMG and gait data were processed in custom Python scripts. Toe‐on and toe‐off events of the right hindlimb were identified from synchronized treadmill video recordings and used to define stance phases during locomotion. Stance duration was calculated as the time between toe‐on and toe‐off events for each stride. Raw EMG signals were baseline‐corrected, bandpass filtered (20–450 Hz), rectified, and smoothed with a root mean‐square (RMS) envelope (20 ms). Strides were defined by consecutive heel contacts, stance was defined as heel contact to the first toe‐off within the stride, and per‐stride stance duration and peak RMS EMG during stance were extracted for each stride.

### NMJ function

CMAPs were assessed as a functional measure of VL NMJ innervation. CMAPs were recorded under the plane of anaesthesia as described above. A 1 cm incision was made in the inguinal region of the affected (right) hindlimb, and the motor branch of the femoral nerve was exposed, taking care to minimize tissue disruption. Nerve temperature was maintained with a feedback temperature control system (TCAT‐2DF Animal Temperature Controller – Dual Feature, Physitemp) and kept moist with sterile saline throughout the procedure. The stimulating electrode was placed proximally along the motor branch of the femoral nerve, while a recording epimysial electrode was positioned distally on the VL. A reference/ground electrode was placed subcutaneously near the base of the tail. A digital electrophysiological system (Nicolet EDX EMG System, Natus) was used to deliver electrical stimuli and record CMAPs. Supramaximal stimulation was applied as single square pulses (0.1 ms duration, 0.5–2.5 mA intensity) at 1 Hz to ensure consistent activation of all motor units. CMAPs were measured at supramaximal current, and CMAP amplitude was calculated by subtracting peak‐to‐peak values of the evoked response (in mV).

### NMJ integrity

Bilateral VLs were collected for the assessment of NMJ integrity. While under anaesthesia, incisions were made in the right and left hindlimbs and bilateral VL muscles were excised. Muscles were fixed in 5% neutrally buffered formalin for 15 min, rinsed with PBS, and flash‐frozen in liquid nitrogen. Samples were stored at −80°C for NMJ morphology assessments via immunohistochemistry.

Longitudinal muscle sections (35–40 µm) from bilateral VL muscles were cut and mounted on glass microscope slides. Slides were then prepped (e.g. air‐dried overnight, washed in methanol 1 × 1 min, then washed in PBS 3 × 5 min) and blocked in 5% goat serum diluted in 0.1% PBS‐Tween 20 (PBS‐T) for 30 min at room temperature. Following blocking, slides were incubated overnight at 4°C with primary antibodies against neurofilament medium chain (NF‐M; 2H3, IgG1, 1:50; DSHB) and synaptic vesicle glycoprotein 2A (SV2, IgG1, 1:50; DSHB) to label axons and pre‐synaptic nerve terminals, respectively. The following day, slides were washed with PBS‐T and incubated overnight at 4°C with secondary antibodies (goat anti‐mouse Alexa Fluor 488; 1:2000; Invitrogen #A‐21121) and co‐stained with alpha‐bungarotoxin (1:2000; Invitrogen #B35451). Following incubation, slides were cover‐slipped with mounting medium (H‐1000, Vector Laboratories, Burlingame, CA, United States). Imaging was subsequently captured at 10× magnification (Keyence BZ‐X800, Keyence Corporation, Osaka, Japan) for analysis. Endplates were classified as innervated when there was colocalization of the NF/SV2 signal with the alpha‐bungarotoxin signal and denervated when there was no overlap of the NF/SV2 and alpha‐bungarotoxin signals. Analyses were conducted by a trained observer (D.A.) blinded to the experimental condition.

### Statistical analysis

All analyses were performed in R (Foundation for Statistical Computing). Data are presented as means ± SD, and alpha was set at 0.05. Outcomes were analysed using paired *t* tests (body composition, WMW, NMJ morphology), two‐way ANOVA (CMAP amplitude) or linear mixed‐effects models (lme4, lmerTest, emmeans; fibre‐level outcomes, stride duration, EMG) depending on the design of each experiment; the specific model for each outcome is described in the Results.

For all mixed‐effects analyses of fibre‐level outcomes, the right limb served as the injured side and the left as the within‐animal contralateral control. Each animal contributed fibre‐level data at a single timepoint only, so Timepoint was treated as a between‐subject factor. A random intercept per animal was included throughout. Limb symmetry was expressed as Limb symmetry index (LSI) = 100 × (affected/contralateral).

Gait data (peak RMS EMG, stance duration) were collected longitudinally at baseline and at 6 h (6H), 3, 7 and 14 days post‐injury. Stride‐level data were filtered to remove non‐physiological values and strides exceeding ±3 SD within each Subject × Timepoint (<1.75% of strides). Mixed‐effects models for gait included random intercepts for Subject and Trial (i.e. treadmill bout). Peak RMS EMG was log‐transformed prior to modelling and contrasts back‐transformed to percentage change *versus* baseline; stance duration was analysed on the raw scale.

For all mixed‐effects models, fixed effects and interactions were tested using Type III F‐tests with Satterthwaite degrees of freedom. *Post hoc* contrasts were derived from estimated marginal means only following significant omnibus effects, with Holm's correction within the relevant contrast family.

NMJ function was assessed using peak‐to‐peak CMAP amplitude and compared between age‐matched healthy controls and 3‐day injured animals using two‐way ANOVA with Injury Status and Sex as factors. NMJ integrity was quantified as the percentage of fully innervated NMJs per animal in the affected and contralateral VL and compared between limbs using paired *t* tests within each sex.

## Results

### Body composition

Body weight declined significantly from baseline to endpoint in both sexes at T7 (females: Δ −11.33 ± 5.09 g, *P* = 0.0028; males: Δ −12.33 ± 3.44 g, *P* = 0.0003). At T14, body weight remained unchanged in females (Δ −5.50 ± 6.72 g, *P* = 0.1012) but increased significantly in males (Δ +21.50 ± 8.60 g, *P* = 0.0017). Lean mass decreased significantly in T7 females (Δ −7.70 ± 3.95 g, *P* = 0.0050), with T7 males showing a trend toward a decrease that did not reach significance (Δ −14.39 ± 14.66 g, *P* = 0.0612). Lean mass increased significantly in T14 males (Δ +12.76 ± 9.49 g, *P* = 0.0216); no change was observed in T14 females (*P* = 0.8923). Fat mass decreased significantly at T7 in both females (Δ −2.79 ± 1.97 g, *P* = 0.0179) and males (Δ −3.88 ± 1.78 g, *P* = 0.0031). At T14, fat mass was unchanged in females (Δ −2.83 ± 5.78 g, *P* = 0.2845) but increased significantly in males (Δ +4.36 ± 1.92 g, *P* = 0.0026). The changes in body composition observed across cohorts were controlled for with the use of the contralateral limb as a within‐subject control.

### WMW

To assess changes in whole‐muscle mass following ACL injury, VL WMW was compared between the affected (right) and contralateral (left) limbs across timepoints in both females and males (Table 1). In uninjured controls (T0), no significant side‐to‐side differences were observed in either females or males. Following ACL injury, significant atrophy of the evident at both T7 and T14 in females (T7: *P* = 0.0235; T14: *P* = 0.0091) and males (T7: *P* = 0.0015; T14: *P* = 4.79 × 10^−5^). WMWs of additional quadriceps muscles are presented in Table A1.

**Table 1:** Vastus lateralis (VL) wet muscle weight (WMW), measured in milligrams (mg), prior to anterior cruciate ligament (ACL) injury for healthy controls (T0) and 7 and 14 day ACL‐injured rats (T7, T14).

Sex	*n*	Left (contralateral) (mean ± SD, mg)	Right (affected) (mean ± SD, mg)	Δ R−L (mean ± SD, mg)	*t* (df)	*P* (raw)	Sig.
T0							
Female	6	789.10 ± 62.53	778.40 ± 56.49	−10.70 ± 48.36	−0.542 (5)	0.6111	ns
Male	6	1462.23 ± 130.53	1509.23 ± 53.73	47.00 ± 103.71	1.110 (5)	0.3175	ns
T7							
Female	6	1040.90 ± 214.81	768.65 ± 71.67	−272.25 ± 207.07	−3.220 (5)	**0.0235**	^*^
Male	6	1405.73 ± 93.87	1180.08 ± 69.21	−225.65 ± 87.59	−6.310 (5)	**0.0015**	^**^
T14							
Female	6	915.85 ± 128.45	781.37 ± 79.41	−134.48 ± 79.90	−4.123 (5)	**0.0091**	^**^
Male	6	1460.95 ± 66.25	1211.48 ± 90.63	−249.47 ± 46.97	−13.009 (5)	**4.79e‐05**	^***^

*Note*: Δ (mg) indicates the difference between left (contralateral) and right (affected) limbs. Raw paired *t* test *P* values are displayed for each comparison. Values are means ± standard deviation. Paired two‐sided *t* test. Left = contralateral limb; Right = affected limb.

Abbreviation: ns, not significant.

*** *P* < 0.001.

** *P* < 0.01.

* *P* < 0.05.

### VL fibre CSA is significantly reduced following ACL injury

**Figure 2:**
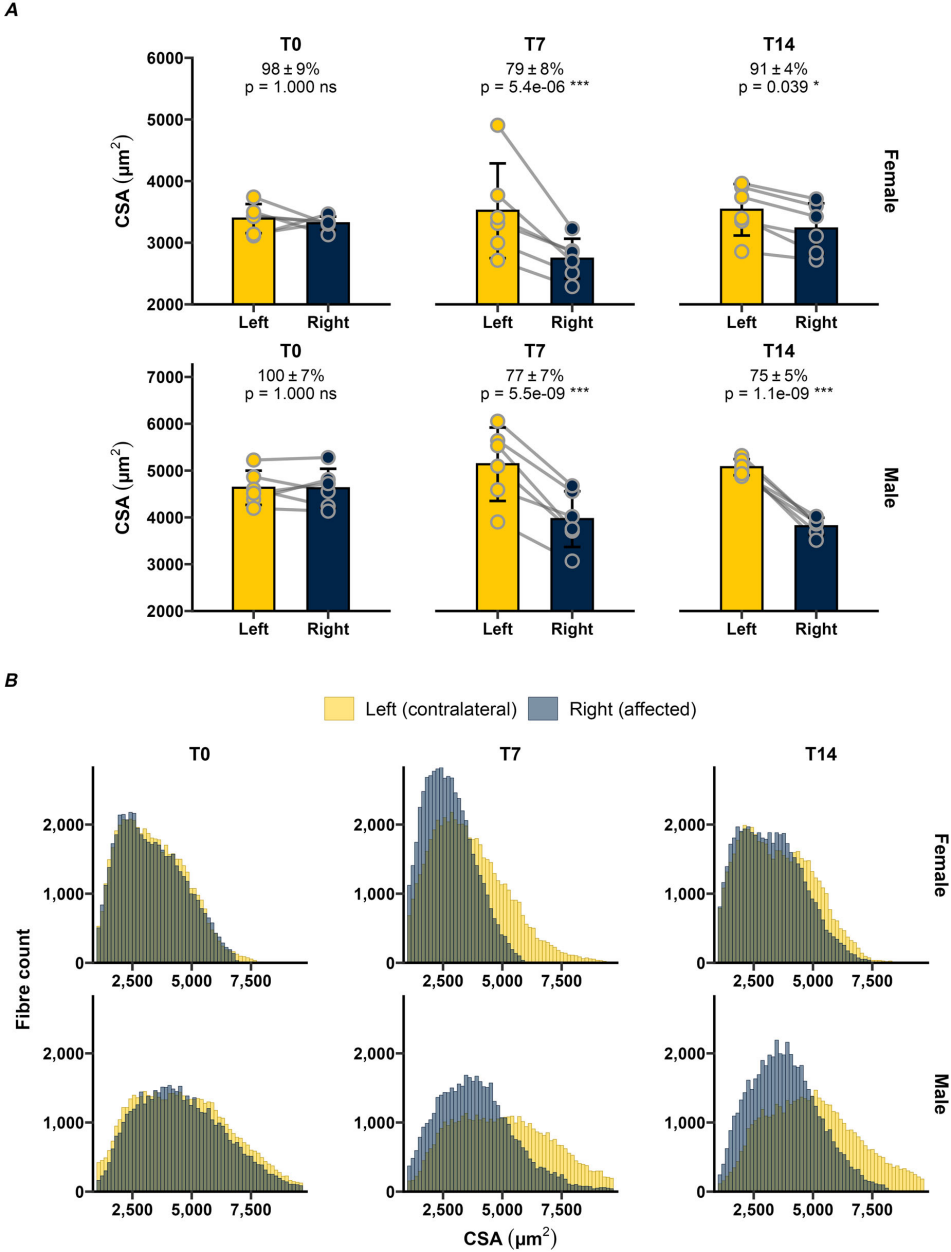
Vastus lateralis (VL) fibre cross‐sectional area (CSA) before and after anterior cruciate ligament (ACL) injury *A*, subject‐level paired comparison: each animal's overall mean (±SD) fibre CSA per limb (pooled across all fibre types) with bars showing across‐animal means ± SD and lines linking the contralateral (yellow) and affected (blue) limbs within each animal at baseline (T0) and 7 and 14 days post‐injury (T7, T14; *n* = 6/group). Limb symmetry index (LSI; affected/contralateral × 100) and Holm‐adjusted *P* values from linear mixed‐model contrasts are displayed above each comparison. *B*, histograms of fibre‐level CSA, with all individual fibres pooled across animals within each sex and timepoint for contralateral (yellow) and affected (blue) limbs. At baseline, CSA was well matched in both sexes (LSI: ∼99%). Following injury, the affected limb (blue) showed reductions in mean fibre CSA at T7 and T14, accompanied by a visible leftward shift in the CSA distribution, consistent with early fibre atrophy.

To characterize the overall effect of ACL injury on VL fibre size, we examined mean fibre CSA pooled across fibre types in the affected limb (right) relative to the contralateral (left) within each cohort. Each timepoint (T0, T7, T14) comprised a separate cohort of animals, with affected and contralateral limbs assessed within each animal. A linear mixed‐effects model with Limb × Sex × Timepoint as fixed effects and animal as a random intercept revealed a highly significant main effect of Limb (F(1,30) = 108.85, *P* = 1.69 × 10^−11^), confirming that fibres in the affected VL were smaller than those of the contralateral limb overall (Fig. 2A). In addition, we observed a significant main effect of Sex (F(1,30) = 80.91, *P* = 5.08 × 10^−10^), with males demonstrating substantially larger VL fibres than females overall, consistent with sexual dimorphism in skeletal muscle size. The Limb × Sex interaction was also significant (F(1,30) = 13.90, *P* = 0.0008), indicating that the magnitude of the between‐limb deficit differed between sexes when collapsed across timepoints. The Limb × Timepoint interaction was likewise significant (F(2,30) = 24.38, *P* = 5.16 × 10^−7^), indicating that the between‐limb deficit evolved over the post‐injury period, prompting examination of within‐timepoint contrasts. Fixed effects accounted for a substantial proportion of variance in fibre CSA (marginal *R* ^2^ = 0.734), with conditional *R* ^2^ increasing to 0.922 once between‐animal variability was included, indicating that Limb, Sex and Timepoint together captured the majority of variance in fibre size. In females at 7 days post‐injury, we observed a substantial deficit in the affected limb (LSI: 79% ± 8%, *P* = 5.4 × 10^−6^), with a smaller but still statistically significant deficit observed in 14‐day injured females (LSI: 91% ± 4%, *P* = 0.039). In males, the affected limb had marked atrophy at 7 days post‐injury (LSI: 77% ± 7%, *P* = 5.5 × 10^−9^) with similar deficits observed in the 14‐day injured cohort (LSI: 75% ± 5%, *P* = 1.1 × 10^−9^). As expected, uninjured control animals (T0) showed no side‐to‐side differences in fibre CSA in either sex (females LSI: 98% ± 9%, *P* = 1.000; males LSI: 100% ± 7%, *P* = 1.000). Fibre‐size distributions (Fig. 2B), generated from pooled fibre‐level data across all animals within each sex and timepoint, reinforced these patterns, as fibres in the affected limb showed a clear leftward shift toward smaller CSA values relative to the contralateral side in both sexes at 7 days. At 14 days, affected fibres underwent a similar leftward shift in males, while the extent of the leftward shift was reduced but still present in the affected fibres of females.

To formally test whether the magnitude of CSA reduction in the affected VL differed between sexes across post‐injury cohorts, we examined the three‐way Limb × Sex × Timepoint interaction. This interaction was significant (F(2,30) = 6.61, *P* = 0.004), indicating that magnitude of atrophy in the affected VL was not consistent across sex and timepoint. While the affected‐limb deficit at 7 days was comparable between sexes, the female and male cohorts diverged at 14 days, with substantially less limb CSA asymmetry in females than males (Fig. 2).

### Reductions in CSA after ACL injury are observed across Type IIa, IIx and IIb fibres

**Figure 3:**
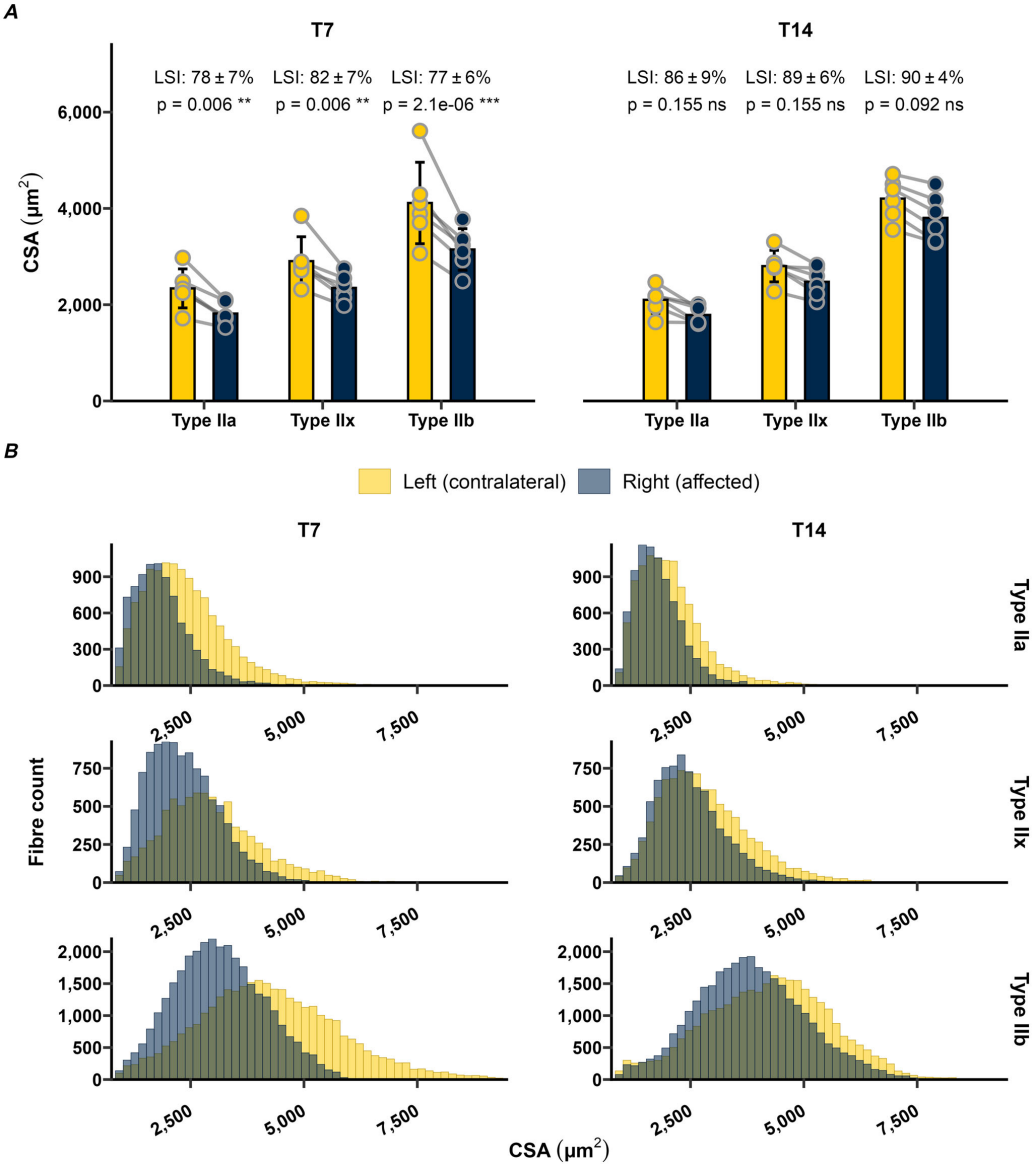
Female vastus lateralis (VL) fibre‐type specific cross‐sectional area (CSA) following anterior cruciate ligament (ACL) injury *A*, subject‐level paired comparison: each animal's overall mean (±SD) fibre CSA per limb for Type IIa, IIx and IIb fibres at 7 and 14 days post‐injury (T7, T14; *n* = 6/group). Type I fibres were excluded due to low prevalence. Limb symmetry index (LSI; affected/contralateral × 100) and Holm‐adjusted *P* values from linear mixed‐model contrasts are displayed above each comparison. *B*, histograms of fibre‐level CSA, with all individual fibres pooled across animals within each sex and timepoint for contralateral (yellow) and affected (blue) limbs for each fibre type. Across all Type II fibre subtypes, the affected limb exhibited significantly reduced mean CSA relative to the contralateral limb, with a corresponding leftward shift in the size distribution indicating global reductions in fibre CSA following ACL injury.

Having established that ACL injury results in significant CSA deficits in the affected VL irrespective of fibre type, we next sought to determine whether this atrophy was driven by selective vulnerability of specific fibre types or reflected a more generalized response across the predominant fibre types assessed in this study (Type IIa, IIx, IIb); Type I fibres were excluded due to their relative sparsity across the dataset. A linear mixed‐effects model (Limb × Fibre Type × Sex × Timepoint; random intercept: animal) revealed a significant main effect of Limb (F(1,100) = 254.1, *P* = 3.3 × 10^−29^), confirming reduced CSA in the injured limb VL. A significant Limb × Fibre Type interaction was also observed (F(2,100) = 6.99, *P* = 0.001), indicating that the magnitude of atrophy varied across fibre types. Fixed effects explained a large proportion of variance in fibre‐type CSA (marginal *R* ^2^ = 0.857; conditional *R* ^2^ = 0.936), indicating that Limb, Fibre Type, Sex and Timepoint combined accounted for the majority of variability in fibre size. Accordingly, *post hoc* comparisons were performed to assess the extent of atrophy within each fibre type. At 7 days post‐injury, females exhibited significant CSA reductions in the affected limb across all predominant fibre types, including Type IIa (LSI: 78% ± 7%, *P* = 0.006), Type IIx (LSI: 82% ± 7%, *P* = 0.006) and Type IIb fibres (LSI: 77% ± 6%, *P* = 2.1 × 10^−6^; Fig. 3A). At 14 days, however, these deficits were mitigated and were no longer statistically significant (Type IIa LSI: 86% ± 9%, *P* = 0.155; Type IIx LSI: 89% ± 6%, *P* = 0.155; Type IIb LSI: 90% ± 4%, *P* = 0.092), consistent with the attenuation observed for pooled CSA in females (Fig. 2). The fibre‐size histograms (Fig. 3B), comprising all individual fibres pooled across animals within each fibre type (i.e. not subject‐averaged), corroborated these findings: the affected limb showed a clear leftward shift toward smaller CSA across all three fibre types at T7, with a more muted shift at T14.

**Figure 4:**
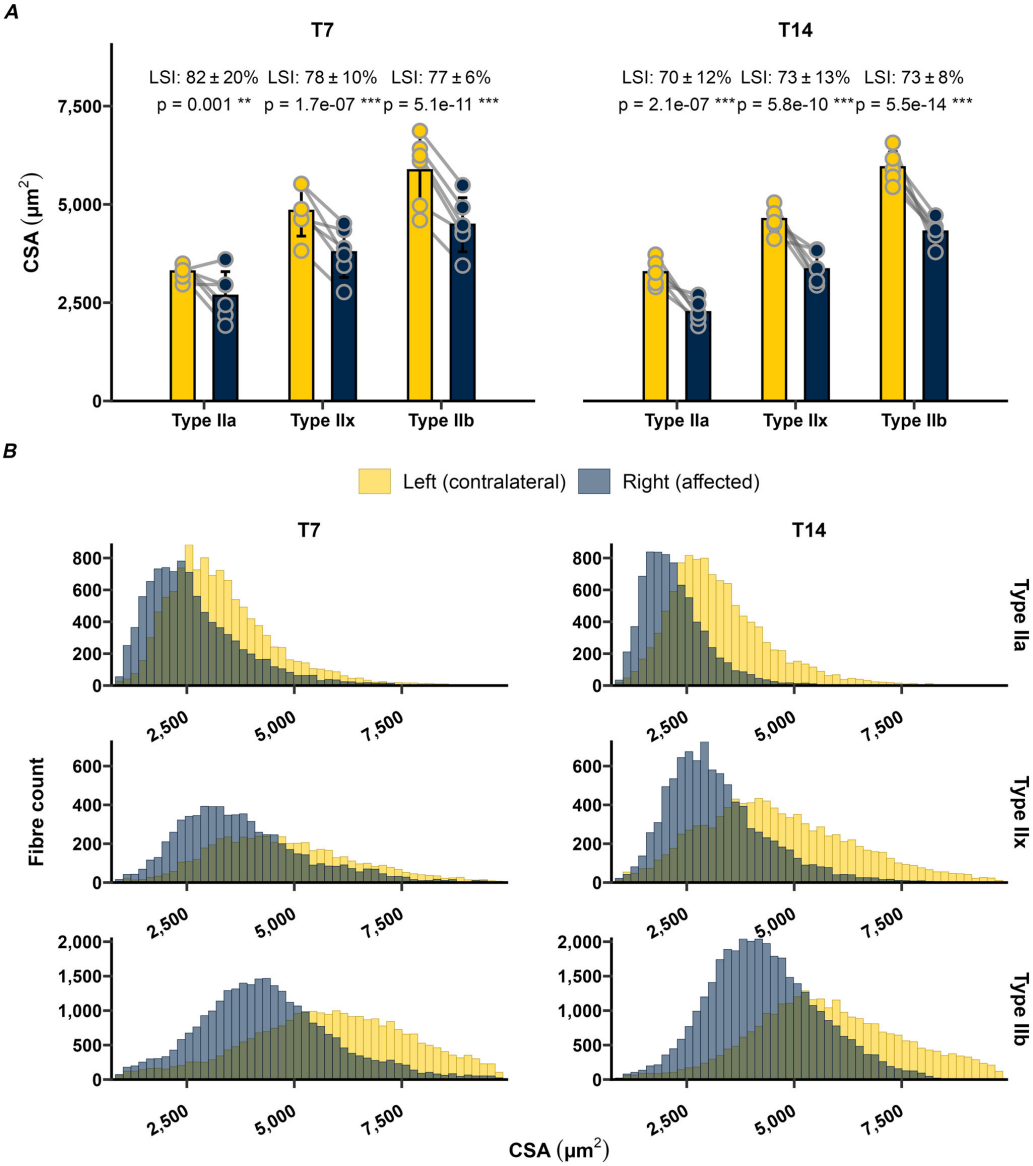
Male vastus lateralis (VL) fibre‐type specific cross‐sectional area (CSA) following anterior cruciate ligament (ACL) injury *A*, subject‐level paired comparison: each animal's overall mean (±SD) fibre CSA per limb for Type IIa, IIx and IIb fibres at 7 and 14 days post‐injury (T7, T14; *n* = 6/group). Type I fibres were excluded due to low prevalence. Limb symmetry index (LSI; affected/contralateral × 100) and Holm‐adjusted *P* values from linear mixed‐model contrasts are displayed above each comparison. *B*, histograms of fibre‐level CSA, with all individual fibres pooled across animals within each sex and timepoint for contralateral (yellow) and affected (blue) limbs for each fibre type. Type IIa, IIx and IIb fibres showed significantly reduced CSA in the affected limb at both timepoints. A leftward shift in the size distribution was evident across fibre types, consistent with a progressive, fibre‐type independent reduction in CSA following ACL injury.

In males, ACL injury produced significant CSA reductions across all three predominant fibre types at both post‐injury timepoints. At 7 days post‐injury, significant deficits were evident in Type IIa (LSI: 82% ± 20%, *P* = 0.001), Type IIx (LSI: 78% ± 10%, *P* = 1.7 × 10^−7^) and Type IIb fibres (LSI: 77% ± 6%, *P* = 5.1 × 10^−11^). At 14 days, significant CSA reductions were maintained across all predominant fibre types (Type IIa LSI: 70% ± 12%, *P* = 2.1 × 10^−7^; Type IIx LSI: 73% ± 13%, *P* = 5.8 × 10^−10^; Type IIb LSI: 73% ± 8%, *P* = 5.5 × 10^−14^; Fig. 4A). The fibre‐size histograms (Fig. 4B) illustrate this, with leftward shifts in the affected limb maintained across all three fibre types at both timepoints. Taken together with the pattern observed in females (Fig. 3), these findings indicate that ACL injury is associated with VL atrophy affecting Type IIa, IIx and IIb fibres in both sexes during the acute post‐injury phase (T7). At 14 days, a similar degree of atrophy is evident across these fibre types in males relative to T7, whereas females do not exhibit significant atrophy in any of the predominant fibre types.

To determine whether these patterns reflected proportional or absolute differences in fibre‐type vulnerability, we modelled the deficit in the affected limb on both relative (ΔCSA% = (affected – contralateral) / contralateral × 100) and absolute (affected – contralateral, µm^2^) scales as by fibre type within each sex and timepoint. The absolute deficit showed a strong main effect of fibre type (F(2,40) = 14.96, *P* = 1.4 × 10^−5^), driven by the larger absolute losses in Type IIb fibres. In contrast, the same model fit to relative percentage loss showed no main effect (F(2,40) = 0.30, *P* = 0.745), indicating that the extent of fibre atrophy is not different when accounting for relative differences in size across fibre types. The greater absolute decline observed in Type IIb fibres therefore reflects their inherently larger baseline size compared with Type IIx and IIa fibres rather than a differential atrophic response (Figs. 3 and 4). Fixed effects explained a comparatively modest proportion of variance in relative (%) CSA loss (marginal *R* ^2^ = 0.273; conditional *R* ^2^ = 0.649) but a larger proportion of variance in absolute CSA deficit (marginal *R* ^2^ = 0.480; conditional *R* ^2^ = 0.757). Collectively, these findings indicate that although the magnitude of atrophy varies somewhat among individual animals, ACL injury induces a proportionally uniform reduction in fibre CSA across all fibre types, suggesting that no single fibre type is disproportionately vulnerable to atrophy following injury in this model.

### VL fibre number is unchanged after ACL injury

We next sought to determine whether fibre number loss also contributed to the observed reductions in whole‐muscle atrophy as indicated by WMW (Table 1). Given that reductions in fibre number are most commonly associated with denervation (Kostrominova, 2022), and that NCAM, a marker of neuromuscular distress, has been reported following ACL injury (Fry et al., 2017; Hunt et al., 2022), we examined whether total fibre number differed between the affected and contralateral VL at each post‐injury timepoint. A linear mixed‐model examining total fibre number with Side (affected vs. contralateral), Timepoint (T0, T7, T14) and Sex as fixed effects, and a random intercept and Side slope per subject, revealed no significant main effects of Side (F(1,30) = 1.56, *P* = 0.222), Timepoint (F(2,30) = 0.26, *P* = 0.775) or Sex (F(1,30) = 1.81, *P* = 0.189). Critically, the Side × Timepoint interaction was not significant (F(2,30) = 0.30, *P* = 0.741), indicating that limb asymmetry in total fibre number did not differ between limbs at T0, T7 and T14 following injury. Descriptive statistics for left (contralateral) and right (affected) fibre counts are presented in Table 2.

**Table 2:** Fibre number per cross‐section in the left (contralateral) and right (affected) vastus lateralis (VL) muscles in healthy control (T0) and 7‐ and 14‐day ACL‐injured (T7, T14) male and female rats (*n* = 6/group).

Sex	*n* (paired)	Left (contralateral)	Right (affected)	Mean Δ (R−L)	*P*	Sig.
T0						
Female	6	8667.5 ± 1198.5	8604.2 ± 1368.7	−63.3 ± 1130.6	0.896	ns
Male	6	9215.5 ± 856.7	8444.8 ± 870.2	−770.7 ± 1071.5	0.138	ns
T7						
Female	6	9373.8 ± 1654.0	8934.2 ± 1450.8	−439.7 ± 1173.5	0.401	ns
Male	6	7789.8 ± 1024.4	7603.7 ± 920.9	−186.2 ± 1189.2	0.717	
T14						
Female	6	8892.8 ± 1514.3	8391.2 ± 597.1	−501.7 ± 1613.2	0.481	ns
Male	6	8275.0 ± 1159.3	8699.7 ± 1528.7	424.7 ± 1131.4	0.400	ns

*Note*: Values are means ± standard deviation.

Abbreviation: ns, non‐significant.

### Affected VLs exhibit a Type IIb‐shifted fibre‐type composition by 14 days post‐injury

After characterizing changes in fibre CSA and number, we also sought to address a related question that has received long‐standing interest and speculation in the field: whether ACL injury alters the MyHC phenotype of VL fibres. Although the fibre‐type spectrum in the rat VL differs substantially from that of humans, most notably due to the predominance of Type IIb fibres in rodents, which are essentially absent in human skeletal muscle, the model still provides an opportunity to comprehensively evaluate injury‐induced shifts in fibre‐type composition. Additionally, the present fibre‐type analysis specifically pertained to Type IIa, IIx and IIb fibres, with Type I fibres excluded due to their low abundance across samples. To examine whether ACL injury results in a fibre‐type transition, we modelled fibre‐type proportions in the affected *versus* contralateral VL using a linear mixed‐effects model with Limb × Fibre Type × Sex × Timepoint as fixed effects and animal as a random intercept. Fixed effects (Limb, Fibre Type, Sex, Timepoint, and their interactions) explained the substantial majority of variance in fibre‐type proportion (marginal *R* ^2^ = 0.902), driven predominantly by the large main effect of Fibre Type (F(2,180) = 959.85, *P* = 9.6 × 10^−97^). As none of the Sex × Limb interaction terms reached significance in this model (Limb × Sex *P* = 0.936; Limb × Fibre Type × Sex *P* = 0.090; Limb × Sex × Timepoint *P* = 0.974; Limb × Fibre Type × Sex × Timepoint *P* = 0.268), male and female animals were pooled for the *post hoc* contrasts comparing affected with contralateral VLs to maximize statistical power. This differs from the sex‐stratified analysis used for fibre CSA, where significant sex interaction (Limb × Sex *P* = 0.0008, Limb × Sex × Timepoint *P* = 0.004) necessitated separate analysis by sex. The pooled model revealed a significant Limb × Fibre Type interaction (F(2,180) = 4.49, *P* = 0.012), indicating that side‐to‐side differences in fibre‐type proportions were present across fibre types. The Limb × Fibre Type × Timepoint interaction did not reach statistical significance (F(4,180) = 2.30, *P* = 0.061) and the timepoint‐specific contrasts below are therefore reported to characterize the significant Limb × Fibre Type effect rather than as confirmation of a statistically distinct temporal trajectory.

As expected, fibre‐type composition did not differ between limbs in uninjured controls (Type IIa Δ = +0.4%, *P* = 0.876; Type IIx Δ = −3.1%, *P* = 0.264; Type IIb Δ = +2.2%, *P* = 0.417). At 7 days post‐injury, no fibre type showed a significant shift in proportion (Type IIa Δ = −4.8%, *P* = 0.080; Type IIx Δ = +4.0%, *P* = 0.147; Type IIb Δ = +1.3%, *P* = 0.644), although Type IIa exhibited a non‐significant trend toward reduced proportion in the affected limb. At 14 days post‐injury, however, the affected VL exhibited a significantly greater proportion of Type IIb fibres relative to the contralateral limb (Δ = +7.3%, *P* = 0.009), accompanied by a non‐significant trend toward reduced Type IIa proportion (Δ = −4.8%, *P* = 0.081) and no detectable change in Type IIx proportion (Δ = −2.3%, *P* = 0.402; Table 3). Collectively, these findings indicate that fibre‐type composition in the affected VL trends toward a Type IIb‐enriched profile by 14 days post‐injury; however, because the Limb × Fibre Type × Timepoint interaction was not statistically significant, this result should be interpreted as exploratory and hypothesis‐generating rather than evidence of a progressive transition.

**Table 3:** Proportion of Type IIa, Type IIx and Type IIb fibres in the left (contralateral) and right (affected) vastus lateralis (VL) muscles of uninjured controls (T0) and 7‐ and 14‐day ACL‐injured (T7, T14) pooled across male and female rats (*n* = 12/group). Type I fibres were excluded due to their relatively low prevalence across samples.

Fibre type	*n*	Left (contralateral)	Right (affected)	Mean Δ (R−L) [95% CI]	*P* (raw)	*P* (Holm)	Sig.
T0							
Type IIa	12	19.8 ± 5.1	20.3 ± 6.9	0.4 [−5.0, 5.9]	0.876	0.876	ns
Type IIx	12	21.5 ± 3.7	18.4 ± 8.9	−3.1 [−8.5, 2.4]	0.264	0.793	ns
Type IIb	12	57.9 ± 6.6	60.1 ± 10.6	2.2 [−3.2, 7.7]	0.417	0.835	ns
T7							
Type IIa	12	23.9 ± 7.2	19.1 ± 5.4	−4.8 [−10.3, 0.6]	0.080	0.240	ns
Type IIx	12	13.7 ± 4.7	17.7 ± 5.3	4.0 [−1.4, 9.4]	0.147	0.294	ns
Type IIb	12	61.0 ± 10.7	62.3 ± 8.5	1.3 [−4.2, 6.7]	0.644	0.644	ns
T14							
Type IIa	12	20.5 ± 4.5	15.7 ± 3.4	−4.8 [−10.3, 0.6]	0.081	0.162	ns
Type IIx	12	18.9 ± 4.7	16.6 ± 6.1	−2.3 [−7.7, 3.1]	0.402	0.402	ns
Type IIb	12	59.9 ± 7.6	67.2 ± 6.6	7.3 [1.8, 12.7]	0.009	**0.027**	*

*Note*: Values are means ± SD. Mean Δ represents the mean affected ‐ contralateral difference in fibre‐type proportion. Raw and Holm‐adjusted *P* values are shown for left *versus* right comparisons within each timepoint and fibre type. Statistical significance was set at the alpha level = 0.05. The bold value in the table indicates the *P* < 0.05 threshold. *P < 0.05.

Abbreviation: ns, non‐significant.

### VL activation and stance duration are altered during treadmill walking after ACL injury

To contextualize the observed fibre‐level changes within the broader response to ACL injury, we longitudinally assessed limb loading and quadriceps activity via stance duration and VL EMG, respectively, in a separate cohort of rats during a standardized treadmill challenge performed at a constant pace (16 m/min) prior to and at serial timepoints following injury.

**Figure 5:**
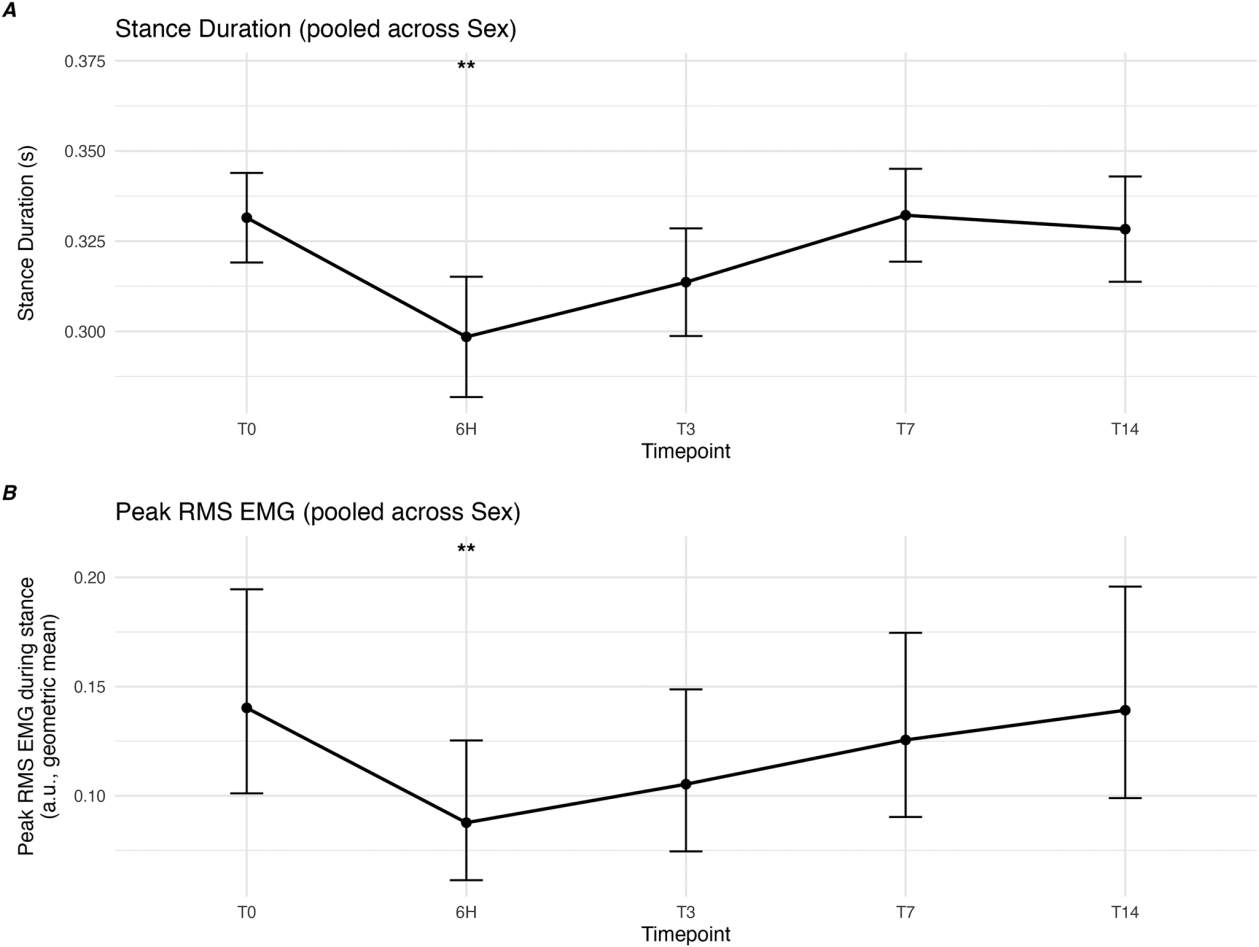
Stride‐level linear mixed‐model estimates of stance duration and peak RMS EMG across time following anterior cruciate ligament ACL injury Estimated marginal means (±95% confidence intervals) derived from linear mixed models are shown for stance duration (*A*) and peak root mean‐square electromyographic (RMS EMG) amplitude during stance (*B*), prior to injury (T0) and at 6 h (6H), 3, 7 and 14 days post‐injury. Models included fixed effects of Sex, Timepoint and their interaction, with random intercepts for Subject and Trial. Because the Sex × Timepoint interaction was not significant for either outcome, estimated marginal means are shown pooled across sex. Peak RMS EMG was modelled on the log scale and back‐transformed for visualization as geometric means. Symbols denote model‐estimated means and lines illustrate temporal trajectories within each sex. Asterisks indicate significant *post hoc* comparisons *versus* T0 within sex following Holm's adjustment (*P < 0.05, **P < 0.01).

With respect to stance duration, the linear mixed model revealed a significant main effect of Sex (F(1,15.30) = 16.01, *P* = 0.0011) and Timepoint (F(4,147.59) = 4.94, *P* = 0.0009), with no significant Sex × Timepoint interaction (F(4,147.59) = 0.81, *P* = 0.5201). Because the interaction was not significant, *post hoc* contrasts were performed on estimated marginal means pooled across sex. Stance duration was reduced at 6 h post‐injury relative to baseline (Δ = −0.033 s, *P* = 0.0013), with no significant differences at 3 days (Δ = −0.018 s, *P* = 0.0920), 7 days (Δ = 0.001 s, *P* = 1.000) or 14 days (Δ = −0.003 s, *P* = 1.000) post‐injury (Fig. 5A). Fixed effects explained a small proportion of variance in stance duration (marginal *R* ^2^ = 0.075), with the conditional *R* ^2^ increasing to 0.157 after accounting for subject‐ and trial‐level random effects.

For peak RMS EMG during stance, the linear mixed model revealed a significant main effect of Timepoint (F(4,142.73) = 3.71, *P* = 0.0066), with no main effect of Sex (F(1,15.32) = 1.19, *P* = 0.2921) and no Sex × Timepoint interaction (F(4,142.73) = 0.25, *P* = 0.9081). As with stance duration, *post hoc* contrasts were therefore performed pooled across sex. The significant Timepoint main effect was driven by an acute reduction in VL EMG activity at 6 h post‐injury, with peak RMS EMG reduced by 37.5% relative to baseline (*P* = 0.0013). A similar, non‐significant trend toward reduced EMG amplitude was observed at 3 days (−24.9%, *P* = 0.0501), with no differences at 7 days (−10.5%, *P* = 0.5822) or 14 days (−0.8%, *P* = 0.9465) post‐injury (Fig. 5B). Fixed effects only explained a small proportion of variance in log EMG (marginal *R* ^2^ = 0.062); with the conditional *R* ^2^ increasing to 0.511 after accounting for subject‐ and trial‐level random effects.

### NMJ function is preserved early after ACL injury

To determine whether changes in NMJ function preceded the observed fibre atrophy, we assessed CMAP amplitude as a proxy for neuromuscular transmission integrity in a separate cohort of rats at 3 days post‐injury relative to a cohort of uninjured healthy controls. CMAP amplitude did not significantly change at 3 days post‐ACL injury as assessed by two‐way ANOVA, with no main effect of injury status (F(1,20) = 0.544, *P* = 0.469), no main effect of sex (F(1,20) = 3.105, *P* = 0.093), and no injury status × sex interaction (F(1,20) = 0.245, *P* = 0.626). Within‐sex comparisons and descriptive statistics are presented in Table 4. Females demonstrated a mean reduction of 3.52 mV (95% CI: −6.82, −0.21) at 3 days post‐injury, whereas males had no change (95% CI: −13.65, 12.27), though 3‐day injured males demonstrated greater inter‐individual variability.

**Table 4:** Compound muscle action potential (CMAP) amplitude (mV) in the vastus lateralis (VL) of healthy control (HC) and 3‐day ACL‐injured (T3) male and female rats.

Sex	*n* (HC)	*n* (T3)	HC	T3	Mean Δ (T3−HC)	95% CI (T3−HC)
Female	6	6	50.34 ± 2.09	46.82 ± 2.91	−3.52	[−6.82, −0.21]
Male	6	6	53.95 ± 5.89	53.26 ± 12.15	−0.69	[−13.65, 12.27]

*Note*: Values are means ± standard deviation with the alpha level = 0.05.

### VL atrophy is not preceded by overt peripheral denervation early after ACL injury

**Figure 6:**
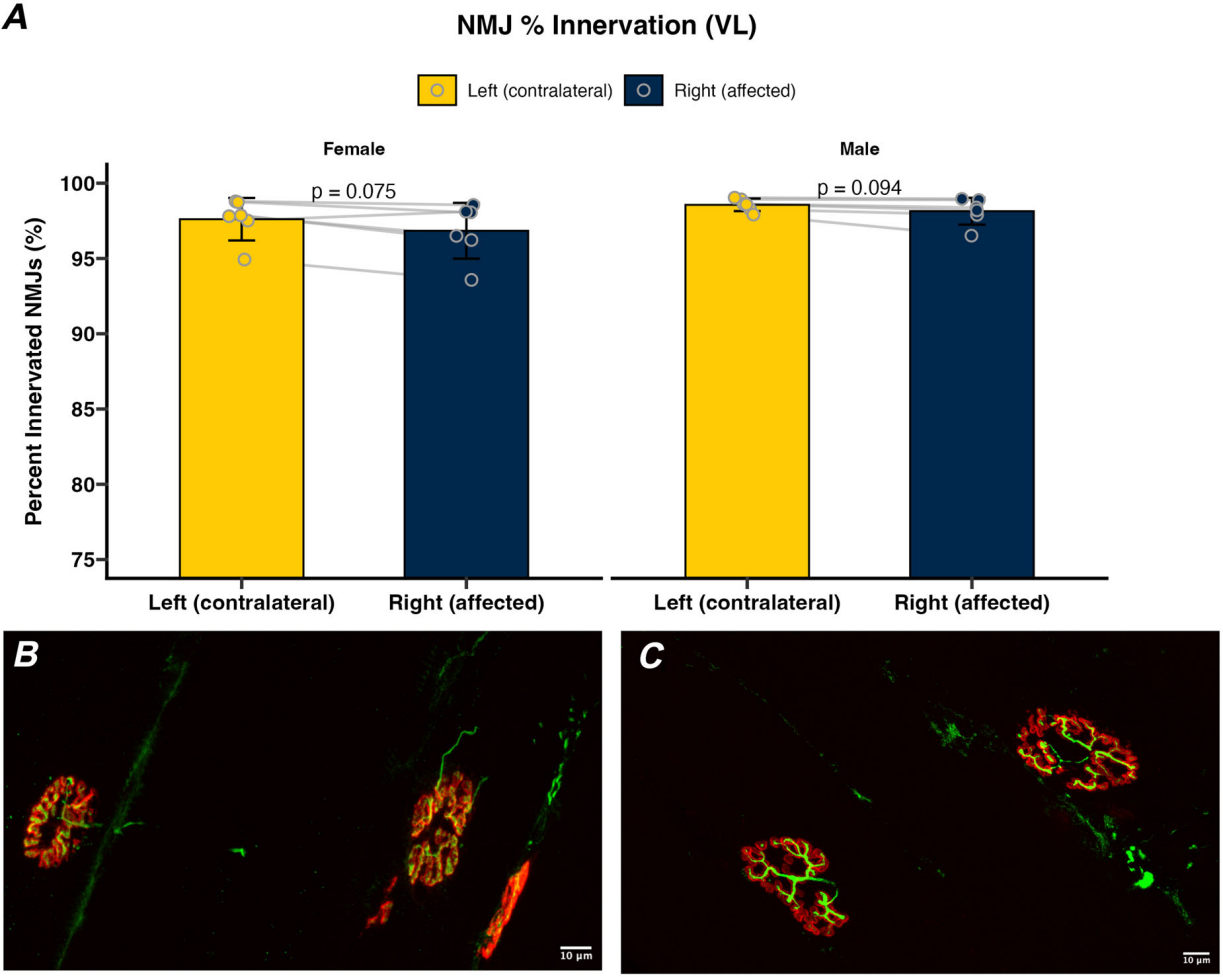
Neuromuscular junction innervation (NMJ) in the vastus lateralis (VL) at 3 days post‐anterior cruciate ligament (ACL) injury *A*, percentage innervated neuromuscular junctions (NMJs) in VL are shown for contralateral control (yellow) and affected (blue) VLs in female and male rats (*n* = 6/group). *A*, bars represent means ± SD, while points denote individual animals and connecting lines indicate paired comparisons between limbs. Paired *t* tests revealed no significant differences in NMJ innervation between contralateral and affected limbs in either females (*P* = 0.075) or males (*P* = 0.094). Representative 25× confocal images of NMJs from contralateral (*B*), and affected (*C*) VLs. NMJs with overlap of pre‐synaptic terminal and neurofilament (green; SV2/NF‐M) with endplate (red; α‐bungarotoxin) were classified as innervated.

To complement the CMAP findings with a more direct assessment of NMJ integrity, immunofluorescent analysis of longitudinal muscle sections was performed on contralateral and affected VLs at 3 days post‐injury to quantify the proportion of innervated NMJs at the individual fibre level. We evaluated approximately 1000 NMJs per muscle on average for contralateral and affected muscles for each animal (*n* = 6 per sex; 24 muscles total; ∼24,000 NMJs evaluated overall). Mean NMJ counts were: male contralateral control VL = 1125 ± 350, male affected VL = 1075 ± 330, female contralateral control VL = 1112 ± 285, and female affected VL = 891 ± 239. The percentage of innervated NMJs did not differ between contralateral and affected VLs in males (98.56% ± 0.42% vs. 98.14% ± 0.89%) or females (97.61% ± 1.41% vs. 96.84 ± 1.84%; Fig. 6), although a trend toward a marginal decrease (<1%) in both sexes is noted.

## Discussion

Quadriceps atrophy is a well‐established consequence of ACL injury and a known risk factor for long‐term declines in joint health, physical activity and quality of life (Ajuied et al., 2014; Birchmeier et al., 2020; Mall et al., 2014; Mather et al., 2013). This persistent atrophy has motivated clinicians and researchers alike to identify more effective therapies to restore quadriceps size and strength after injury. A fundamental step in that process is understanding the aetiology of whole‐muscle quadriceps atrophy. Prior work has not determined to what degree changes in quadriceps size after ACL injury can be attributed to decreases in fibre CSA and/or loss of fibre number, and whether alterations at the NMJs contribute to these changes. The purpose of this study was to utilize whole‐muscle sampling to thoroughly evaluate changes in: (1) fibre CSA, number and fibre‐type distribution, and (2) NMJ integrity and function in the VL following ACL injury in a preclinical model.

Our findings indicate that whole‐muscle atrophy after ACL injury is driven primarily by substantial reductions in fibre CSA rather than loss of muscle fibres. At 3 days post‐injury, before the onset of measurable CSA reductions, we did not detect a significant change in NMJ integrity and function. Within the limitations of the early timepoint and the morphological and electrophysiological outcomes assessed here, the absence of overt NMJ disruption argues against frank denervation as the primary trigger of the significant atrophy observed in this model.

### Early quadriceps atrophy after ACL injury is driven by reductions in fibre CSA

Utilizing a rodent model of non‐invasive ACL injury combined with whole‐muscle histological analysis, the present study quantified changes in muscle fibre CSA, fibre number and fibre‐type distribution following injury. By 7‐day post‐injury, mean fibre CSA was markedly reduced in both males (−23% ± 7%) and females (−21% ± 8%), demonstrating rapid structural remodelling of the quadriceps muscle (Fig. 2). Additionally, reductions in CSA were observed across all three predominant fibre types (Types IIa, IIx and IIb), indicating a gross atrophic response during the early post‐injury period (Figs. 3 and 4).

These findings contrast with biopsy‐based observations in ACL‐injured patients, where greater fibre CSA deficits have been reported in females compared with males (Keeble, Owen et al., 2026; Owen et al., 2024) and where fibre type‐specific atrophy has been described (Keeble, Owen et al., 2026; Latham et al., 2024; Leszczynski et al., 2021; Noehren et al., 2016). These discrepancies likely reflect differences in whether surgical reconstruction was performed, the timing of assessment, and the measurement resolution between models. Human studies are typically conducted weeks to months after injury or reconstruction, whereas the present study isolates the early effects of ACL injury in an ACL‐deficient preclinical model with high temporal and spatial resolution. In doing so, the present model provides a controlled assessment of the direct effects of injury, whereas clinical studies capture this process within a more complex context that includes surgical reconstruction and subsequent recovery (De la Fuente et al., 2025; Thomas et al., 2016). Notably, although the initial atrophic response was similar between sexes (T7 Female LSI: 79% ± 8%, T7 Male LSI: 77% ± 7%), we observed a divergence between males and females during the later phase, with females demonstrating less severe asymmetries in fibre CSA (T14 Female LSI: 91% ± 4%), whereas males exhibited similar deficits to those observed at T7 (T14 Male LSI: 75% ± 5%, Fig. 2). This pattern suggests that sex‐related differences may appear in the trajectory of early recovery rather than in the onset of atrophy. This interpretation is supported by prior work in the same preclinical model, which demonstrated sex‐specific divergence in autophagic flux markers during the 7–14‐day post‐injury window, indicative of less efficient clearance of damaged cellular substrates at the timepoint when CSA recovery diverges (Noh et al., 2024). Importantly, these findings reflect the intrinsic muscle response to ACL injury, prior to the additional demands introduced by surgical reconstruction. As such, the present results may be most applicable to the early post‐injury period, including pre‐operative decision making or patients who elect to remain ACL‐deficient, whereas clinical studies incorporating reconstruction provide important context for how these responses may be altered following surgery.

The discrepancy in findings may also be explained by the relatively small regions of muscle sampled in human biopsies, where estimates of CSA are known to be sensitive to sample size, with variability increasing when fewer than 150–200 fibres and when less than three samples are obtained (Horwath et al., 2021; Nederveen et al., 2020; Van de Casteele et al., 2024). In contrast, the present analysis quantified fibre CSA across entire muscle cross‐sections with more than 8000 fibres per section during the early post‐injury period in a controlled preclinical model. It is also worth noting that female rodents are known to engage in more cage activity relative to their male counterparts (Russell, 1973), although the extent to which differences in early limb use contribute to these sex‐related recovery patterns remains unclear.

To further define the structural basis of this atrophy, we next evaluated whether these reductions in muscle size were accompanied by changes in fibre number. The use of whole‐muscle sampling in our preclinical model facilitated the first comprehensive evaluation of changes in fibre number following ACL injury. Using this approach, we demonstrated that VL fibre number was not significantly altered by ACL injury, with similar fibre number between injured and contralateral VLs at both 7 and 14 days after injury (Table 2). By distinguishing between these two potential contributors to whole‐muscle atrophy, the present work provides direct structural evidence that early muscle loss after ACL injury is driven by reductions in fibre CSA rather than loss of muscle fibres.

### Early quadriceps atrophy occurs in the absence of overt NMJ disruption

The absence of overt NMJ disruption at 3 days post‐injury considered alongside the well‐described neural consequences of ACL injury, including early spinal reflexive inhibition and subsequent reductions in corticospinal and supraspinal drive, is consistent with a centrally mediated inhibitory process resulting in diminished quadriceps activation (Lepley & Lepley, 2021; Luc‐Harkey et al., 2017; Pietrosimone et al., 2022; Snyder‐Mackler et al., 1994). This interpretation is further supported by temporal changes in quadriceps EMG activity and stance duration during gait irrespective of sex (Fig. 5), indicative of reduced volitional activation and limb‐loading early after injury. Together, these findings suggest that reduced muscle activation, rather than overt disruption of peripheral innervation, is likely responsible for the initiation of muscle atrophy through canonical signalling pathways and depressed protein synthesis (Bodine, 2013), leading to reductions in fibre CSA without fibre loss.

This interpretation is consistent with prior work demonstrating upregulation of hallmark atrogenes in the quadriceps during a similar post‐injury period in both ACL‐injured patients and rodent models of ACL injury (Brightwell et al., 2023; Delfino et al., 2013; Durigan et al., 2014; Keeble et al., 2023). Notably, recent multi‐omics work has also identified modest upregulation of denervation‐responsive signalling following ACL reconstruction (Keeble, Gonzalez et al., 2026), although these changes occur in the context of surgical intervention and may not reflect the isolated response to injury alone. Therefore, the data presented herein provide early evidence against gross denervation as the initiating signal that precedes quadriceps atrophy. Although NMJ structure and function were not assessed beyond the early post‐injury timepoint, EMG quadriceps amplitude has been shown to improve over the weeks to months following ACL injury (Shanbehzadeh et al., 2017), a trajectory more consistent with central inhibition than with progressive peripheral denervation.

### Fibre‐type distribution suggests a shift toward a more glycolytic contractile phenotype following ACL injury

The whole‐muscle sampling approach used in this study also enabled evaluation of changes in VL fibre‐type distribution following ACL injury. While prior work has observed potential shifts in fibre phenotype after injury in small populations of muscle fibres (Davi et al., 2022; Flück et al., 2018; Graham et al., 2024; Noehren et al., 2016), the present study utilized whole‐muscle cross‐sections to examine a potential phenotype transition in the affected VL during the first 14 days post‐injury. Specifically, we observed a non‐significant trend toward a reduction in the relative abundance of Type IIa fibres at 7 days, with a significant increase in Type IIb fibres at 14 days (Table 3), a pattern consistent with a fibre‐type transition toward a more glycolytic muscle profile following injury; however, the Limb × Fibre Type × Timepoint interaction underlying this apparent time course did not reach statistical significance (*P* = 0.061), and this temporal interpretation should therefore be considered preliminary. This pattern is similar to fibre‐type transitions observed under conditions of reduced muscle activation and loading, where diminished contractile activity promotes a shift towards a more glycolytic muscle (Sharlo et al., 2021; Shenkman, 2016). Together, these findings provide early, exploratory preclinical evidence that fibre‐type remodelling toward an increased proportion of Type IIb fibres is present in the VL after ACL injury in this model, likely as a response to reduced contractile activity and mechanical demand; confirmation of the temporal progression of this shift will require adequately powered follow‐up studies.

### Clinical implications

The distinction between atrophy mediated by decreased CSA versus diminished fibre number has clinical importance. Fibre CSA reductions, likely driven by reduced muscle activation and the downstream suppression of protein synthesis and activation of canonical atrophy pathways, are, in principle, more amenable to reversal through interventions that restore contractile activity and mechanical stress to the quadriceps. In contrast, preventing or reversing denervation and associated fibre loss requires therapies designed to promote reinnervation, such as functional electrical stimulation, which target fundamentally different adaptations (Kern & Carraro, 2020; Pieber et al., 2015). Thus, identifying decreased fibre CSA, rather than fibre loss, as the primary consequence of ACL injury highlights the potential for targeted neuromuscular or mechanical therapies to preserve and or restore muscle size in the post‐injury period. These findings also align with the current standard‐of‐care clinical strategies that aim to minimize effusion and pain, restore range of motion and promote early weightbearing, all of which may help mitigate inhibition and unloading‐associated changes in muscle fibre size and phenotype (Sonnery‐Cottet et al., 2019). Hence, these findings suggest that the standard‐of‐care approach is directionally appropriate in its aim to address fibre‐level atrophy after ACL injury. However, given the persistent deficits in quadriceps size observed in many ACL‐injured patients (De la Fuente et al., 2025; Ito et al., 2025) and the added influence of surgery on muscle adaptation, rehabilitation strategies must evolve to more effectively treat quadriceps atrophy.

### Limitations

The present study is not without its limitations. First, NMJ integrity and function were evaluated at a single early timepoint (3 days post‐injury) to capture neural inhibition prior to measurable atrophy. As such, the present findings cannot exclude the possibility of delayed or progressive NMJ remodelling. In addition, NMJ integrity was assessed using a binary classification (innervated vs. denervated) to enable large‐scale whole‐muscle sampling, which did not capture NMJs with partial overlap or more subtle features of NMJ remodelling. However, the functional significance of partial overlap at the NMJ remains uncertain as the large safety factor means that incomplete overlap does not necessarily compromise transmission (Wood & Slater, 2001). Second, although whole‐muscle sampling enabled comprehensive evaluation of fibre CSA, number and distribution, estimates of fibre number remain sensitive to section location and quality. All VL muscles were sectioned at the mid‐section of the muscle belly by a single trained technician to minimize variability, and fibre counts in control muscles were similar (Table 1), suggesting that sectioning variability had marginal influence on between‐limb comparisons. Third, fibre‐type classification relied on immunohistochemistry in which Type IIx fibres were inferred through negative staining. Although a primary antibody targeting Type IIx exists, it shares the same IgM isotype as the Type IIb antibody, precluding their simultaneous use as both would be detected by the same anti‐IgM secondary antibody. As a result of the negative IIx staining, hybrid fibre populations could not be reliably classified and were excluded from analysis. Fourth, we acknowledge that the predominant fibre types in rat VL (IIa, IIx, IIb) are more glycolytic than those typically observed in human VLs (I, IIa, IIx). Although the specific fibre‐type spectrum slightly differs between species, the directionality of phenotype shifts observed in this model, namely reductions in more oxidative fibres (e.g. IIa) and increases in more glycolytic fibres (e.g. IIb), remains translationally relevant. In humans, a comparable transition would be expected to manifest as reductions in Type I/IIa fibres with relative increases in IIx fibres, rather than the IIb increase observed in this model. Additionally, these analyses were limited to the VL muscle. While the VL was selected for its translational relevance to human biopsy studies and comparability with prior work in this model, caution is warranted when extrapolating these findings to the other quadriceps muscles, which may exhibit distinct responses to ACL injury. With respect to statistical interpretation, a further limitation relates to model complexity as several outcomes were modelled with higher‐order interactions (up to four‐way, e.g. Limb × Fibre Type × Sex × Timepoint) at modest sample sizes (*n* = 6/sex/group), which are likely underpowered to detect true higher‐order effects. We were conservative in interpreting these terms and restricted *post hoc* comparisons to the level supported by significant model terms in addition to reporting marginal and conditional *R* ^2^ for each model as a more robust indicator of fit. Finally, stance duration and EMG amplitude were used as proxies of limb loading and quadriceps activation. Because cage activity was not monitored, they should be interpreted as indirect indicators of global muscle activation and loading.

## Conclusion

These findings demonstrate that early quadriceps remodelling after ACL injury is characterized by widespread reductions in fibre CSA without overt evidence of peripheral denervation. By resolving these adaptations at the whole‐muscle level, this work clarifies the structural basis of early quadriceps dysfunction after ACL injury.

## Additional information

## Competing interests

None declared.

## Author contributions

All experiments were performed in the Comparative Orthopaedic Laboratory at the University of Michigan School of Kinesiology. L.S., L.K.L. and P.C.D contributed to the conception and design of the work. L.S., P.C.D., H.W.H., D.A., S.N., L.V., L.D. and B.B contributed to the acquisition of data. L.S. and K.G contributed to the processing and analysis of data. All authors contributed to drafting and critically revising the manuscript for important intellectual content. All authors approved the final version of the manuscript, agree to be accountable for all aspects of the work in ensuring that questions related to the accuracy or integrity of any part of the work are appropriately investigated and resolved, and confirm that all persons designated as authors qualify for authorship and that all those who qualify for authorship are listed.

## Funding

This work was supported by National Institute of Arthritis and Musculoskeletal and Skin Diseases Grant R01AR081235 (to L.K.L.) and F31AR087153 (to L.S.).

## Generative AI statement

In accordance with Wiley's generative artificial intelligence (GAI) policy and the position statement of the Committee on Publication Ethics, the authors disclose the following use of GAI tools in the preparation of this manuscript. Claude (Anthropic; model: claude‐sonnet‐4‐6) was used to assist in refining written language and improving clarity and readability of the manuscript text. Additionally, Claude (Anthropic; model: claude‐sonnet‐4‐6) was used to assist in the revision and debugging of R Markdown code used for the generation of figures and tables. All AI‐assisted content was reviewed, verified and edited by the authors, who take full responsibility for the accuracy and integrity of the final manuscript. No AI tool was used for data collection, interpretation of results, or in any capacity that constitutes authorship.

## Supporting information


Peer Review History

## Data Availability

The data that support the findings of this study are archived and available upon reasonable request to the corresponding author. Raw immunofluorescence images used for fibre typing, generating composite erosion files, and the associated Python processing pipeline are archived and available upon request. Similarly, raw NMJ morphology data, CMAP recordings, and gait/stance duration measurements, along with their corresponding processing scripts, are archived and available upon request. No data from this study have been deposited in a public repository.

## Methods

### Agreement analyses

Agreement between manual fibre‐type labels and the automated classifier was evaluated on ten 10× images using only fibres for which both manual and automated labels were available. Analyses were restricted to the primary fibre‐type classes (Type I, Type IIa, Type IIx, Type IIb); fibres lacking a valid label in either source were excluded.

Classification agreement was assessed using a confusion matrix, comparing automated predictions to manual labels (reference standard). From this matrix, overall accuracy was calculated as the proportion of fibres correctly classified. Agreement beyond chance was quantified using Cohen's kappa (κ).

To evaluate classification performance across fibre types, class‐specific recall (sensitivity) was calculated as the proportion of manually labelled fibres correctly identified by the automated classifier. Confusion matrices and summary agreement metrics are presented descriptively.

To evaluate performance across fibre types, precision, recall (sensitivity), and F1 scores were calculated for each class using the manual labels as the reference standard. Precision was defined as the proportion of fibres predicted to belong to a given class that were correctly classified (true positive / (true positive + false positive)), whereas recall (sensitivity) represented the proportion of manually labelled fibres correctly identified by the automated classifier (true positive / (true positive + false negative)). The F1 score was calculated as the harmonic mean of precision and recall (2 × precision × recall / (precision + recall)) to summarize overall classification performance for each fibre type. In addition, macro‐averaged and prevalence‐weighted averages of these metrics were calculated across fibre types.

## Results

### Wet muscle weight

Within‐subject comparisons revealed significant reductions in affected‐limb WMW for several quadriceps’ muscles following ACL injury (Table A1). In females at T7 and at T14, affected‐limb WMW was significantly lower than the contralateral limb for VL and rectus femoris (RF), whereas vastus medialis (VM) and vastus intermedius (VI) did not differ between limbs. In males at T7, affected‐limb WMW was significantly reduced for the VL, RF and VM, with no difference observed for the VI. At T14, significant asymmetry persisted for the VL, RF, and VM, and a significant increase in affected‐limb WMW was observed for the VI relative to the contralateral limb.

**Table A1:** Paired comparisons of left (contralateral) and right (affected) wet muscle weight (WMW, in mg) was performed for each muscle, sex and timepoint using paired *t* tests.

Muscle	*n*	Left (contralateral) Mean ± SD	Right (affected) Mean ± SD	Δ (mg) Mean ± SD	%Δ vs. contralateral	LSI ± SD	*P* (raw)
Female – T0							
Vastus lateralis	6	789.1 ± 62.5	778.4 ± 56.5	−10.7 ± 48.4	−1.2% ± 6.2%	98.8 ± 6.2	0.6111
Rectus femoris	6	809.7 ± 40.3	818.2 ± 52.6	8.6 ± 20.4	1.0% ± 2.5%	101.0 ± 2.5	0.3514
Vastus medialis	6	363.1 ± 32.6	368.4 ± 32.9	5.2 ± 17.8	1.5% ± 5.0%	101.5 ± 5.0	0.5029
Vastus intermedius	6	95.3 ± 11.1	98.6 ± 23.3	3.2 ± 23.5	3.9 ± 24.0%	103.9 ± 24.0	0.7514
Female – T7							
Vastus lateralis	6	1040.9 ± 214.8	768.6 ± 71.7	−272.2 ± 207.1	−24.3% ± 13.1%	75.7 ± 13.1	**0.0235**
Rectus femoris	6	962.2 ± 124.0	847.0 ± 100.0	−115.2 ± 44.3	−11.8% ± 3.5%	88.2 ± 3.5	**0.0014**
Vastus medialis	6	387.3 ± 53.1	333.8 ± 75.1	−53.5 ± 67.5	−13.5% ± 16.6%	86.5 ± 16.6	0.1099
Vastus intermedius	6	106.0 ± 22.8	118.7 ± 29.2	12.6 ± 30.7	14.4% ± 33.9%	114.4 ± 33.9	0.3611
Female – T14							
Vastus lateralis	6	915.9 ± 128.5	781.4 ± 79.4	−134.5 ± 79.9	−14.2% ± 6.8%	85.8 ± 6.8	**0.0091**
Rectus femoris	6	874.4 ± 102.8	795.3 ± 93.5	−79.1 ± 26.5	−9.0% ± 2.6%	91.0 ± 2.6	**0.0007**
Vastus medialis	6	410.6 ± 69.0	358.8 ± 48.4	−51.8 ± 49.7	−11.6% ± 11.3%	88.4 ± 11.3	0.0510
Vastus intermedius	6	103.5 ± 35.4	106.0 ± 31.8	2.4 ± 35.8	7.3% ± 40.0%	107.3 ± 40.0	0.8735
Male – T0							
Vastus lateralis	6	1462.2 ± 130.5	1509.2 ± 53.7	47.0 ± 103.7	3.8% ± 7.7%	103.8 ± 7.7	0.3175
Rectus femoris	6	1577.6 ± 101.6	1588.0 ± 97.9	10.3 ± 26.0	0.7% ± 1.7%	100.7 ± 1.7	0.3758
Vastus medialis	6	674.5 ± 51.5	694.1 ± 68.3	19.7 ± 57.7	3.1% ± 8.4%	103.1 ± 8.4	0.4411
Vastus intermedius	6	160.7 ± 53.8	205.8 ± 64.6	45.2 ± 58.7	38.6% ± 56.2%	138.6 ± 56.2	0.1179
Male – T7							
Vastus lateralis	6	1405.7 ± 93.9	1180.1 ± 69.2	−225.6 ± 87.6	−15.9% ± 5.4%	84.1 ± 5.4	**0.0015**
Rectus femoris	6	1614.8 ± 153.4	1380.4 ± 121.5	−234.4 ± 56.1	−14.4% ± 2.6%	85.6 ± 2.6	**0.0002**
Vastus medialis	6	746.8 ± 51.1	587.3 ± 33.0	−159.5 ± 52.9	−21.1% ± 6.1%	78.9 ± 6.1	**0.0007**
Vastus intermedius	6	182.1 ± 30.2	173.3 ± 11.6	−8.8 ± 22.2	−3.3% ± 11.8%	96.7 ± 11.8	0.3779
Male – T14							
Vastus lateralis	6	1461.0 ± 66.3	1211.5 ± 90.6	−249.5 ± 47.0	−17.1% ± 3.5%	82.9 ± 3.5	**4.79e‐05**
Rectus femoris	6	1482.1 ± 90.2	1315.7 ± 75.3	−166.5 ± 66.1	−11.1% ± 4.2%	88.9 ± 4.2	**0.0016**
Vastus medialis	6	634.3 ± 81.9	524.8 ± 46.6	−109.6 ± 98.4	−15.9% ± 14.6%	84.1 ± 14.6	**0.0415**
Vastus intermedius	6	146.3 ± 28.3	203.6 ± 40.5	57.3 ± 25.3	40.3 ± 20.7%	140.3 ± 20.7	**0.0026**

*Note*: Paired two‐sided *t* test. Left = contralateral limb; Right = affected limb. Δ = Right − Left. %Δ = (Right − Left) / Left × 100. LSI (Limb Symmetry Index) = (Right / Left) × 100. Bold *P* values indicate *P* < 0.05. Δ indicates the right–left difference (mg), %Δ vs. left indicates the percentage difference relative to the contralateral limb, and limb symmetry index (LSI) was calculated as 100 × (R/L). Values are means ± standard deviation with alpha level = 0.05.

### Manual fibre‐typing validation

Agreement between manual and automated fibre‐type classification was high. Across 4751 manually annotated fibres, the automated classifier achieved an overall accuracy of 98.3% with excellent agreement beyond chance (Cohen's κ = 0.969) (Fig. A1). Class‐specific performance was similarly strong, with recall values ranging from 0.974 to 1.000 across fibre types. Misclassifications occurred primarily between Type IIx and Type IIb fibres, whereas classification of Type I and Type IIa fibres demonstrated near‐perfect agreement. Misclassification between Type IIx and Type IIb fibres is somewhat expected as Type IIx fibres are identified by the absence of staining in the myosin heavy‐chain channels and therefore are more susceptible to bleed‐through from Type IIb fibres, the predominant channel. Precision values were 1.000 across all fibre types, yielding F1 scores between 0.987 and 1.000 (Table A2).

**Table A2:** Classification performance was evaluated across 4751 manually labelled fibres.

Fibre type	Precision	Recall (sensitivity)	F1 score
Type I	1.000	1.000	1.000
Type IIa	1.000	0.974	0.987
Type IIb	1.000	0.988	0.994
Type IIx	1.000	0.974	0.987
**Macro average**	**1.000**	**0.984**	**0.992**
**Weighted average**	**1.000**	**0.982**	**0.991**

*Note*: Precision represents the proportion of predicted fibres belonging to a given class that were correctly classified, whereas recall (sensitivity) represents the proportion of manually labelled fibres correctly identified by the automated classifier. The F1 score represents the harmonic mean of precision and recall. Macro averages represent the unweighted mean across fibre types, and weighted averages account for differences in true fibre‐type prevalence.
