# Supplementary material for: Structural determinants of quadriceps atrophy following ACL injury: Evidence for fibre atrophy without fibre loss or overt peripheral denervation
Source: J Physiol. 2026 Aug 23;604(17):7287–312. doi: 10.1113/JP291436 (PMC13532943; doi:10.1113/JP291436)
Supplement: Supplementary file 1 [file TJP-604-7287-s001.pdf]

The Journal of Physiology

# Structural Determinants of Quadriceps Atrophy Following ACL Injury: Evidence for Fibre Atrophy Without Fibre Loss or Overt Peripheral Denervation

Luke Stoneback, Brianna Buchanan, Hadi W Hussein, Dania Ali, Sairub Naaz, Keegan Gustafson, Leah Vaikutis, Lucas Damouni, Peter CD Macpherson, and Lindsey K Lepley
**DOI: 10.1113/JP291436**

*Corresponding author(s): Lindsey Lepley (llepley@umich.edu)*

*The following individual(s) involved in review of this submission have agreed to reveal their identity: Casper Soendenbroe (Referee #2)*



## Review Timeline:

| | |
|---|---|
| Submission Date: | 30-Mar-2026 |
| Editorial Decision: | 01-May-2026 |
| Revision Received: | 12-Jun-2026 |
| Editorial Decision: | 08-Jul-2026 |
| Revision Received: | 13-Jul-2026 |
| Accepted: | 28-Jul-2026 |

*Senior Editor: Paul Greenhaff*

*Reviewing Editor: Mathew Piasecki*


## Transaction Report:

(Note: With the exception of the correction of typographical or spelling errors that could be a source of ambiguity, letters and reports are not edited. Depending on transfer agreements, referee reports obtained elsewhere may or may not be included in this compilation. Referee reports are anonymous unless the Referee chooses to sign their reports.)

Re: JP-RP-2026-291436 "**Structural Determinants of Quadriceps Atrophy Following ACL Injury: Evidence for Fibre Atrophy Without Fibre Loss or Peripheral Denervation**" by Luke Stoneback, Brianna Buchanan, Hadi W Hussein, Dania Ali, Sairub Naaz, Keegan Gustafson, Leah Vaikutis, Lucas Damouni, Peter CD Macpherson, and Lindsey K Lepley

Dear Dr Lepley,

Thank you for submitting your manuscript to The Journal of Physiology. It has been assessed by a Reviewing Editor and by 2 expert referees and we are pleased to tell you that it is potentially acceptable for publication following satisfactory major revision.

Please advise your co-authors of this decision as soon as possible.

The referee reports are copied at the end of this email.

Please address all the points raised and incorporate all requested revisions or explain in your Response to Referees why a change has not been made. We hope you will find the comments helpful and that you will be able to return your revised manuscript within 2 months. If your article is NOT for a Special Issue, you may have 9 months to revise. If you require an extension, please contact journal staff: jp@physoc.org. Please note that this letter does not constitute a guarantee for acceptance of your revised manuscript.

Your revised manuscript should be submitted online using the link in your Author Tasks: Link Not Available. This link is accessible via your account as Corresponding Author; it is not available to your co-authors. If this presents a problem, please contact journal staff (jp@physoc.org). Image files from the previous version are retained on the system. Please ensure you replace or remove any files that are being revised.

If you do not wish to submit a revised version of your manuscript, you must inform our journal staff (jp@physoc.org) or reply to this email to request withdrawal. Please note that a manuscript must be formally withdrawn from the peer review process at one journal before it may be submitted to another journal.

TRANSPARENT PEER REVIEW POLICY: To improve the transparency of its peer review process, The Journal of Physiology publishes online as supporting information the peer review history of all articles accepted for publication. Readers will have access to decision letters, including Editors' comments and referee reports, for each version of the manuscript, as well as any author responses to peer review comments. Referees can decide whether or not they wish to be named on the peer review history document.

ABSTRACT FIGURES: Authors are expected to use The Journal's premium BioRender account to create/redraw their Abstract Figures. Information on how to access this account is here: https://physoc.onlinelibrary.wiley.com/journal/14697793/biorender-access.

This will enable Authors to create and download high-resolution figures. If authors have used the free BioRender service, they can use the instructions provided in the link above to download a high-resolution version suitable for publication.

The link provided should only be used for the purposes of this submission. Authors will be charged for figures created on this account if they are not related to this manuscript submission.

LANGUAGE EDITING AND SUPPORT FOR PUBLICATION: If you would like help with English language editing, or other article preparation support, Wiley Editing Services offers expert help, including English Language Editing, as well as translation, manuscript formatting, and figure formatting at www.wileyauthors.com/eeo/preparation. You can also find resources for Preparing Your Article for general guidance about writing and preparing your manuscript at www.wileyauthors.com/eeo/prepresources.

RIGOUR AND REPRODUCIBILITY: We recommend authors consult The Journal's Rigour and Reproducibility requirements to ensure that their manuscript is suitable for publication: https://physoc.onlinelibrary.wiley.com/pb-assets/hub-assets/physoc/documents/TJP-Rigour-and-Reproducibility-Requirements.pdf

REVISION CHECKLIST:

Check that your Methods section conforms to journal policy: https://jp.msubmit.net/cgi-bin/main.plex?form_type=display_requirements#methods.

Check that data presented conforms to the statistics policy: https://jp.msubmit.net/cgi-bin/main.plex?form_type=display_requirements#statistics.

Upload a full Response to Referees file. To create your 'Response to Referees': copy all the reports, including any comments from the Senior and Reviewing Editors, into a Microsoft Word, or similar, file and respond to each point, using font or background colour to distinguish comments and responses and upload as the required file type.

Please upload two versions of your manuscript text: one with all relevant changes highlighted and one clean version with no changes tracked. The manuscript file should include all tables and figure legends, but each figure/graph should be uploaded as separate, high-resolution files.

You may also upload:

- 'Potential Cover Art' for consideration as the issue's cover image
- Appropriate Supporting Information (Video, audio or data set: see https://jp.msubmit.net/cgi-bin/main.plex?form_type=display_requirements#supp).

We look forward to receiving your revised submission.

If you have any queries, please reply to this email and we will be pleased to advise.

Yours sincerely,

Paul Greenhaff
Senior Editor
The Journal of Physiology

----------------

REQUIRED ITEMS

- Author photo and profile. First or joint first authors are asked to provide a short biography (no more than 100 words for one author or 150 words in total for joint first authors) and a portrait photograph. These should be uploaded and clearly labelled together in a Word document with the revised version of the manuscript. See Information for Authors for further details.

- Your manuscript must include a complete Additional Information section, including competing interests; funding; author contributions and acknowledgements.

- The Journal of Physiology funds authors of provisionally accepted papers to use the premium BioRender site to create high resolution schematic figures. Follow this link and enter your details and the manuscript number to create and download figures. Upload these as the figure files for your revised submission. If you choose not to take up this offer, we require figures to be of similar quality and resolution. If you are opting out of this service to authors, state this in the Comments section on the Detailed Information page of the submission form. The link provided should only be used for the purposes of this submission. Authors will be charged for figures created on this premium BioRender account if they are not related to this manuscript submission.

- Please upload separate high-quality figure files via the submission form.

- Please ensure that any tables are editable and in Word format, and wherever possible, embedded in the article file itself.

- Papers must comply with the Statistics Policy: https://jp.msubmit.net/cgi-bin/main.plex?form_type=display_requirements#statistics.

In summary:

- If n {less than or equal to} 30, all data points must be plotted in the figure in a way that reveals their range and distribution. A bar graph with data points overlaid, a box and whisker plot or a violin plot (preferably with data points included) are acceptable formats.

- If n > 30, then the entire raw dataset must be made available either as supporting information, or hosted on a not-for-profit repository, e.g. FigShare, with access details provided in the manuscript.

- 'n' clearly defined (e.g. x cells from y slices in z animals) in the Methods. Authors should be mindful of pseudoreplication.

- All relevant 'n' values must be clearly stated in the main text, figures and tables.

- The most appropriate summary statistic (e.g. mean or median and standard deviation) must be used. Standard Error of the Mean (SEM) alone is not permitted.

- Exact p values must be stated. Authors must not use 'greater than' or 'less than'. Exact p values must be stated to three significant figures even when 'no statistical significance' is claimed.

- Please include an Abstract Figure file and an Abstract Figure legend. An appropriate figure legend, which should not exceed 150 words in length, should be included in the main manuscript file. The Abstract Figure is a piece of artwork designed to give readers an immediate understanding of the research and should summarise the main conclusions. If possible, the image should be easily 'readable' from left to right or top to bottom. It should show the physiological relevance of the manuscript so readers can assess the importance and content of its findings. Abstract Figures should not merely recapitulate other figures in the manuscript. Please try to keep the diagram as simple as possible and without superfluous information that may distract from the main conclusion(s). Abstract Figures must be provided by authors no later than the revised manuscript stage and should be uploaded as a separate file during online submission labelled as File Type 'Abstract Figure'. Please also ensure that you include the figure legend in the main article file. All Abstract Figures should be created using BioRender. Authors should use The Journal's premium BioRender account to export high-resolution images. Details on how to use and access the premium account are included as part of this email.

- Please ensure that all figures and tables have a title and legend, and that they have been cited within the main article text.

----------------

EDITOR COMMENTS

Please also see 'Required Items' above.

Reviewing Editor: Methods Details:
Include detail on the method of euthanasia.

Comments to the Author (Required):
Your manuscript has been reviewed by two experts in the field. While the overall assessment is positive, several important concerns must be addressed before the manuscript can be considered for publication. Among the most important of these are issues related to the statistical approach and the interpretation of statistical outcomes, as highlighted in detail by Reviewer #2.

In particular, the rationale for analysing sexes separately should be clarified, as this results in relatively small sample sizes for several measures ($n = 6$). If this approach is considered more appropriate than combining sexes and including sex as a factor in a mixed-model analysis, a clear justification should be provided.

Additionally, care should be taken to avoid overstating the interpretation of statistical findings. For example, the conclusion that NMJs remain intact is not fully supported by the data. Statistical outcomes of $p = 0.075$ and 0.094 from bilateral comparisons with $n = 6$ (Figure 4) do not justify interpreting the conditions as equivalent.

These concerns, along with those raised by the reviewers, should be thoroughly addressed in any revised submission.

Senior Editor:

Comments for Authors to ensure the paper complies with the Statistics Policy (Required):
-Exact p-values are not reported (values given as $p < 0.05$ / $p > 0.05$)

Comments to the Author:
Thank you for the manuscript submission to The Journal of Physiology. It has been considered by a reviewing editor and two expert reviewers. Whilst the reviewers are largely favourable about the manuscript, it is clear that the statistical approach employed by the authors and the language used to interpret statistical outcomes requires significant overhaul. As an example of the later, the Reviewing Editor has highlighted that the the authors repeatedly describe a lack of statistical significance as a 'preservation'; but failure to observe a statistical difference is a long way form describing something as unchanged, particularly when considering the statistical approach employed.

From a housekeeping perspective the method of termination (euthanasia) at the end of experimentation is not stated in the

Methods section and Figure 4 appears not to be cited in the text.

The authors are therefore requested to address all feedback provided and make major revisions to the manuscript for it to be considered further. We look forward to receiving the revised version.

-----------------

REFEREE COMMENTS

Referee #1:

The manuscript by Stoneback et al. examines the impact of anterior cruciate ligament (ACL) injury in a rat model on the vastus lateralis (VL) muscle. ACL injury was induced in 16-week old male and female Long Evans rats and the impact on the VL muscle was examined histologically at 3, 7 and 14 days post injury by measuring muscle mass, fiber cross-sectional area and fiber number. In addition, NMJ innervation and compound muscle action potential were measured at 3 days post injury. The results showed that early atrophy of the VL following ACL injury was related to loss of fiber cross-sectional and not fiber number. Further, atrophy was not induced by denervation, but rather decreased loading and neural activation. This was a week executed and designed study. The results are clearly presented and interpreted.

No major concerns.

Minor corrections:

line 78" word missing in sentence, "show":

line 734: spelling "promote"

Referee #2:

Dear Stoneback et al.,

I have reviewed your manuscript with interest. The study addresses an important question, uses generally solid methods, and provides data that, in my view, broadly support the interpretation that muscle loss following ACL rupture is driven primarily by fibre atrophy rather than overt fibre loss. Overall, I am largely aligned with the main direction of the manuscript.

That said, I think there are several areas where the interpretation currently goes beyond what the data can support, and I also have some concerns regarding the statistical approach and aspects of data presentation. I list these below for your consideration.

1. Statistical approach

My main concern relates to the statistical framework. The statistics section is very long, but rather than conveying rigor, it gives the impression that different datasets were handled using different approaches without a sufficiently clear rationale.

You state that multiple t-tests were conducted independently by sex and time point and justify this based on "planned hypotheses." However, unless this analysis plan was pre-registered or documented in a dated protocol, that justification is

not persuasive. Given that sex and time are both factors in the design, the more appropriate analytical framework would generally be an ANOVA or a linear mixed model. This is especially important for datasets presented side by side within the same graph (for example, Figure 2), where the presentation implies a unified statistical test. This issue appears in multiple datasets and should be addressed consistently.

Related to this, if a linear mixed model shows no significant interaction, then post hoc testing aimed at identifying differential changes between groups over time is not justified. This appears relevant for several outcomes, including Figure 5, and should be reconsidered.

2. Interpretation of neural involvement / NMJ findings

I think the Discussion currently overstates the conclusion that "NMJ integrity and function were preserved." Some of these measures were assessed only at day 3, whereas other outcomes were followed through days 7 and 14. In addition, there are trends in the data and a fibre-type shift, which could reasonably be interpreted as evidence of some neural involvement. For that reason, I do not think the current data supports a strong conclusion that neural changes do not contribute. Rather, the data seems to support the narrower conclusion that the observed atrophy is primarily explained by fibre atrophy.

This concern also applies to the statement on lines 684 onward ("The observed reductions in fibre CSA occurred in the context of preserved NMJ integrity and function..."). I would encourage the authors to moderate this language throughout.

3. NMJ morphology analysis may be too limited for the conclusions drawn

Relatedly, I find the NMJ morphology analysis somewhat too coarse relative to the strength of the conclusions. It is common in this field to quantify partially and fully denervated NMJs, fragmentation, and related indices. It is entirely possible that 3 days was not sufficient to increase the number of fully denervated NMJs, while other morphometric features may still have changed. I recommend checking up on the work of Thomas Gillingwater and adjusting the interpretation accordingly.

Also, for the NMJ dataset, the biologically relevant unit is the number of NMJs analyzed per animal, not simply the total number of NMJs analyzed. Please report this more clearly.

Along similar lines, I think the conclusions regarding preserved NMJ integrity/function should be tempered. Denervation-responsive transcripts such as CHRNA1 and CHRND are known to respond rapidly to altered neuromuscular input, and qPCR analysis of such markers may well have revealed evidence of early denervation-related signaling in the intervention group (e.g. PMID: 32268508). For that reason, I do not think the present data justify such a strong conclusion, and I encourage the authors to revise this interpretation accordingly.

4. Rationale for muscle selection

I missed a clear rationale for focusing specifically on the VL muscle, and for restricting the analysis to that muscle alone. This should be explained.

5. Clarity on sample allocation across assays

Although the total number of animals is clearly stated, I found it difficult at times to determine which animals contributed to which assays, and whether the same animals were used across multiple analyses. I think the manuscript would benefit from a clearer overview of sample allocation.

Minor comments

6. Figures and data presentation

• I recommend adding a black outline around yellow circles in the graphs (for example Figure 4), as these are difficult to see clearly in print.

• Figure 1B and similar histograms would benefit from error bars.

• The y-axis on histograms should be labeled.

• Histograms would be more interpretable if shown as the percentage of fibres in each category, rather than absolute counts, since fibre loss (if it had happened) would otherwise reduce counts across bins by definition.

• The statement on lines 495-497 would also be better aligned with field standards if such differences were expressed in relative rather than absolute terms.

7. Sex-specific presentation is inconsistent

I do not understand why the morphological analyses are separated by sex, whereas fibre-type composition is presented with males and females combined. If sex-specific differences are considered important for one outcome, it seems equally plausible that they could matter for the other. This should either be justified or made more consistent.

8. Fibre classification and exclusion criteria need clarification

I have some concern about how "mis-shaped" fibres may have been handled. Fibre shape has recently been shown to be biologically meaningful (PMID: 38123165), and exclusion based on fixed thresholds may therefore bias the analysis if certain conditions disproportionately affect shape. I am not saying this necessarily occurred here, but the manuscript should more clearly explain what was excluded and why.

Relatedly, lines 240-241 require clarification: "Fibres not meeting these dominance criteria were flagged as ambiguous and subject to manual review by a trained observer." Please explain exactly what this means. Hybrid fibres will almost certainly be present, particularly in the treated condition. How were these handled? Were they assigned to the dominant signal, or treated separately? This needs to be transparent.

9. Supplemental material

As per journal policy, supplemental material and appendices are generally not allowed, and in any case some of these items seem too central to remain outside the main paper. The body composition data could likely be incorporated as a table, and A1 (whole-muscle IF example) seems important for interpretation of the main findings. I would strongly encourage the authors to move essential items into the main manuscript.

10. Specific wording / phrasing issues

A few places in the text would benefit from more careful phrasing:

• Lines 70-71: the sentence implying that reductions in fibre CSA reflect activation of canonical atrophy pathways is too narrow. Reduced protein synthesis is also a plausible and, in many disuse settings, central explanation (PMID: 23800384).

• Lines 71-72: the statement about fibre loss being associated with denervation and impaired reinnervation should be rephrased, as these processes may also activate atrophy pathways. The contrast as written is too sharp (PMID: 28546288).

• Line 640: I think the manuscript underplays how different rodent and human fibre-type distributions are. You acknowledge this later, but the issue is substantial and should be reflected more clearly here as well.

• Lines 766-768: the discussion of fibre-type transitions should be toned down, particularly given how debated this remains in humans.

• Line 198: I assume "lif" should be "tif".

11. CMAP data

The overall conclusion that no change occurred in CMAP appears reasonable based on the presented data. However, the male T3 group seems to show a substantially larger SD than the other groups, which raises some concern about either variance assumptions or technical consistency. I think this deserves checking and possibly commenting on.

---

END OF COMMENTS

**SENIOR EDITOR**

**COMMENT:** Thank you for the manuscript submission to The Journal of Physiology. It has been considered by a reviewing editor and two expert reviewers.
**RESPONSE:** We sincerely appreciate the time and effort the Senior Editor, Reviewing Editor, and Reviewers dedicated to evaluating this manuscript. We found the feedback to be thoughtful, constructive, and believe that it has substantially improved the quality of the manuscript. We have revised the manuscript to address the concerns listed herein, with particular attention to the statistical approach and the framing of the findings relative to NMJ morphology and function in the Results and Discussion sections. All revisions are reflected in the updated manuscript.

**COMMENT:** Whilst the reviewers are largely favourable about the manuscript, it is clear that the statistical approach employed by the authors and the language used to interpret statistical outcomes requires significant overhaul. As an example of the later, the Reviewing Editor has highlighted that the the authors repeatedly describe a lack of statistical significance as a 'preservation'; but failure to observe a statistical difference is a long way form describing something as unchanged, particularly when considering the statistical approach employed.
**RESPONSE:** We have made substantial revisions to both the statistical approach and the interpretive language throughout the manuscript. With respect to statistics, we have replaced the previous series of independent t-tests with linear mixed effects models for all primary outcomes (fibre CSA, number, and composition), providing a unified statistical framework that more appropriately accounts for the within-subject, multi-factor structure of the data. With respect to language, we have carefully reviewed the manuscript to remove instances where non-significant findings were described as evidence of "preservation" or equivalence. Where statistical tests did not reach significance, we have reframed conclusions to reflect the absence of a detectable difference rather than implying preservation, consistent with the editors' and Reviewer #2's concerns.

**COMMENT:** Exact p-values are not reported (values given as $p < 0.05$ / $p > 0.05$)**.**
**RESPONSE:** The results sections have been amended to report exact p-values in accordance with the statistics policy.

**COMMENT:** From a housekeeping perspective the method of termination (euthanasia) at the end of experimentation is not stated in the Methods section and Figure 4 appears not to be cited in the text.
**RESPONSE:** We have added the method of euthanasia to the Methods section (lines 162-163) and ensured Figure 4 is cited in the main text.

**REVIEWING EDITOR**

**COMMENT:** Your manuscript has been reviewed by two experts in the field. While the overall assessment is positive, several important concerns must be addressed before the manuscript can be considered for publication. Among the most important of these are issues related to the statistical approach and the interpretation of statistical outcomes, as highlighted in detail by Reviewer #2.

In particular, the rationale for analysing sexes separately should be clarified, as this results in relatively small sample sizes for several measures ($n = 6$). If this approach is considered more appropriate than combining sexes and including sex as a factor in a mixed-model analysis, a clear justification should be provided.

Additionally, care should be taken to avoid overstating the interpretation of statistical findings. For example, the conclusion that NMJs remain intact is not fully supported by the data. Statistical outcomes of $p = 0.075$ and 0.094 from bilateral comparisons with $n = 6$ (Figure 4) do not justify interpreting the conditions as equivalent.

These concerns, along with those raised by the reviewers, should be thoroughly addressed in any revised submission.
**RESPONSE:** We would like to thank the Reviewing Editor for their attention to the manuscript. We agree that the statistical approach and its interpretation needed significant modification and have taken steps to address these shortcomings. First by replacing the previous series of independent t-tests with linear mixed effects models for all primary outcomes (fibre CSA, number, and composition) to more uniformly address questions relative to fibre CSA, composition, and number.

Second, concerning the sex-specific analyses, we have included Sex as a factor in the linear mixed effects models for all fibre-related outcomes. Sex-stratified presentation of post-hoc contrasts was retained where the omnibus LMM revealed significant Limb x Sex or Limb x Sex x Timepoint interactions, as was the case for fibre CSA (Limb x Sex $p = 0.0008$; Limb x Sex x Timepoint $p = 0.004$), indicating that the magnitude and trajectory of the injured-to-contralateral deficit differed meaningfully between males and females. Where no significant Sex or Sex-interaction terms were identified, as was the case for fibre-type composition (Limb x Sex $p = 0.936$; Limb x FibreType x Sex $p = 0.090$; Limb x Sex x Timepoint $p = 0.974$; Limb x FibreType x Sex x Timepoint $p = 0.268$), data were pooled across sex for post-hoc contrasts to maximize statistical power. We believe this approach, retaining sex-stratification where statistically warranted and pooling where it is not, represents a transparent statistical framework that is justified by the data rather than applied uniformly by assumption.

Third, to address Reviewer #2's concerns regarding the interpretation of statistical outcomes, we revised the manuscript to remove cases where non-significant findings were described as evidence of "preservation" or equivalence with respect to NMJ related analyses.

**COMMENT:** Include detail on the method of euthanasia.
**RESPONSE:** Euthanasia specifics have been added as requested (lines 162-163).

**REFEREE #1**

**COMMENT:** The manuscript by Stoneback et al. examines the impact of anterior cruciate ligament (ACL) injury in a rat model on the vastus lateralis (VL) muscle. ACL injury was induced in 16-week old male and female Long Evans rats and the impact on the VL muscle was examined histologically at 3, 7 and 14 days post injury by measuring muscle mass, fiber cross-sectional area and fiber number. In addition, NMJ innervation and compound muscle action potential were measured at 3 days post injury. The results showed that early atrophy of the VL following ACL injury was related to loss of fiber cross-sectional and not fiber number. Further, atrophy was not induced by denervation, but rather decreased loading and neural activation. This was a week executed and designed study. The results are clearly presented and interpreted.
**RESPONSE:** We would like to thank Reviewer #1 for the time and effort spent evaluating the manuscript. We appreciate their feedback and have taken steps to address the points addressed below.

**COMMENT:** line 78" word missing in sentence, "show":
**RESPONSE:** This has been corrected; “show” has been added to the sentence as requested.

**COMMENT:** line 734: spelling "promote"
**RESPONSE:** The spelling of “promote” has been corrected. Thank you!

**REFEREE #2**

**COMMENT:** I have reviewed your manuscript with interest. The study addresses an important question, uses generally solid methods, and provides data that, in my view, broadly support the interpretation that muscle loss following ACL rupture is driven primarily by fibre atrophy rather than overt fibre loss. Overall, I am largely aligned with the main direction of the manuscript.

That said, I think there are several areas where the interpretation currently goes beyond what the data can support, and I also have some concerns regarding the statistical approach and aspects of data presentation. I list these below for your consideration.
**RESPONSE:** We thank Reviewer #2 for their time and constructive feedback of the manuscript, and we believe that addressing these points, as detailed below, has significantly improved the quality of the manuscript.

**COMMENT:** My main concern relates to the statistical framework. The statistics section is very long, but rather than conveying rigor, it gives the impression that different datasets were handled using different approaches without a sufficiently clear rationale. You state that multiple t-tests were conducted independently by sex and time point and justify this based on "planned hypotheses." However, unless this analysis plan was pre-registered or documented in a dated protocol, that justification is not persuasive. Given that sex and time are both factors in the design, the more appropriate analytical framework would generally be an ANOVA or a linear mixed model. This is especially important for datasets presented side by side within the same graph (for example, Figure 2), where the presentation implies a unified statistical test. This issue appears in multiple datasets and should be addressed consistently.
**RESPONSE:** We agree with this concern. To address these points, we substantially revised both the statistical and Results sections to improve consistency, clarity, and alignment between the experimental design and analytical framework. Specifically, we moved away from the prior approach of conducting multiple independent t-tests across sex and time point and instead employed a more unified series of linear mixed effects models to evaluate changes in fibre CSA, fibre number, and fibre type composition.

In addition, the statistical section has been streamlined to provide a clearer high-level overview of the analytical approaches employed across the study, while the specific animal groups, comparisons, and model structures relevant to each dataset are now detailed at the beginning of the corresponding Results sections. We believe this substantially improves readability, transparency, and interpretation of the statistical framework throughout the manuscript.

**COMMENT:** Related to this, if a linear mixed model shows no significant interaction, then post hoc testing aimed at identifying differential changes between groups over time is not justified. This appears relevant for several outcomes, including Figure 5, and should be reconsidered.
**RESPONSE:** We completely agree with the general principle regarding post-hoc testing following non-significant omnibus tests and can confirm that post-hoc testing was only performed following significant main effects or interactions. We note that as it pertains to Figure 5, main effects for timepoint were found for both stance duration and peak RMS EMG (lines 569-579), thus post-hoc testing was appropriate.

**COMMENT:** I think the Discussion currently overstates the conclusion that "NMJ integrity and function were preserved." Some of these measures were assessed only at day 3, whereas other outcomes were followed through days 7 and 14. In addition, there are trends in the data and a fibre-type shift, which could reasonably be interpreted as evidence of some neural involvement. For that reason, I do not think the current data supports a strong conclusion that neural changes do not contribute. Rather, the data seems to support the narrower conclusion that the observed atrophy is primarily explained by fibre atrophy.

This concern also applies to the statement on lines 684 onward ("The observed reductions in fibre CSA occurred in the context of preserved NMJ integrity and function..."). I would encourage the authors to moderate this language throughout.
**RESPONSE:** As suggested, we have revised the Discussion to be more careful regarding the language around NMJ integrity and function, taking note of the methodological and temporal limitations of our experiments (lines 833-838). Thank you for drawing attention to this important limitation.

**COMMENT:** Relatedly, I find the NMJ morphology analysis somewhat too coarse relative to the strength of the conclusions. It is common in this field to quantify partially and fully denervated NMJs, fragmentation, and related indices. It is entirely possible that 3 days was not sufficient to increase the number of fully denervated NMJs, while other morphometric features may still have changed. I recommend checking up on the work of Thomas Gillingwater and adjusting the interpretation accordingly.
**RESPONSE:** We appreciate this point and agree that the NMJ morphology analyses performed in the current study are relatively coarse compared to the broader range of morphometric approaches commonly employed in the field, including assessments of fragmentation, partial denervation, pre-synaptic morphology, and related indices described in the work of Thomas Gillingwater (Jones *et al.*, 2016) and others (Arnold *et al.*, 2014). We also acknowledge that partially denervated NMJs and more subtle alterations in synaptic architecture may precede overt denervation events and therefore could have been present despite the absence of gross denervation at the 3-day timepoint examined here. However, the primary objective of these experiments was specifically to determine whether overt denervation events preceded the substantial reductions in fibre CSA observed by 7 days post-injury. Within that context, the present findings suggest that widespread gross denervation is unlikely to serve as the initiating signal driving the early atrophic response following ACL injury. At the same time, we fully acknowledge that 1) more subtle changes in NMJ structure and/or denervation-responsive signaling may have been present at 3 days post-injury, and 2) more overt denervation-related changes may emerge at later timepoints not evaluated in the current study. We have revised the Discussion throughout to more carefully reflect these limitations and to avoid overstating the conclusions that can be drawn from the present NMJ analyses.

**COMMENT:** Also, for the NMJ dataset, the biologically relevant unit is the number of NMJs analyzed per animal, not simply the total number of NMJs analyzed. Please report this more clearly.
**RESPONSE:** Agreed. The number of NMJs analyzed per muscle (on average) for each cohort is now reported in lines 719-721.

**COMMENT:** Along similar lines, I think the conclusions regarding preserved NMJ integrity/function should be tempered. Denervation-responsive transcripts such as CHRNA1 and CHRND are known to respond rapidly to altered neuromuscular input, and qPCR analysis of such markers may well have revealed evidence of early denervation-related signaling in the intervention group (e.g. PMID: 32268508). For that reason, I do not think the present data justify such a strong conclusion, and I encourage the authors to revise this interpretation accordingly.
**RESPONSE:** We agree that qPCR analysis of denervation-responsive transcripts such as CHRNA1 and CHRND would strengthen these conclusions, and we acknowledge this as a limitation. We would note, however, that our group has previously demonstrated a transient increase in NCAM expression in this model (Hunt et al., 2022), a marker of denervation-related signaling, which did not persist alongside the atrophy observed at later timepoints. While NCAM, CHRNA1 and CHRND transcripts reflect different aspects of denervation-related signaling, this finding provides at least preliminary molecular evidence consistent with the interpretation that gross peripheral denervation is not the primary driver of early atrophy in this model. We have nonetheless revised the discussion to temper the language around NMJ integrity and function, contextualizing these conclusions to the relevant methodological and temporal limitations of the present dataset (addition of Overt in the title, line 814, lines 833-838).

**COMMENT:** I missed a clear rationale for focusing specifically on the VL muscle, and for restricting the analysis to that muscle alone. This should be explained.
**RESPONSE**: The following rationale has now been added to the manuscript. "The VL was selected to facilitate comparison with prior preclinical work in this ACL injury model (Davi et al., 2022; Hunt et al., 2022; Noh et al., 2024) and to enhance translational relevance to human biopsy-based studies (Refs), where the VL is the most commonly sampled quadriceps muscle." Lines 129-132.

**COMMENT:** Although the total number of animals is clearly stated, I found it difficult at times to determine which animals contributed to which assays, and whether the same animals were used across multiple analyses. I think the manuscript would benefit from a clearer overview of sample allocation.
**RESPONSE:** We agree that the allocation was difficult to follow due to the division of cohorts dedicated to independent outcome measures. We have ensured that the animal numbers per cohort are clearly stated in the Animals and Experimental Groups section of the methods, and we have also added more group clarity to Results section to more accurately contextualize the data to the experimental design where appropriate.

**COMMENT:** I recommend adding a black outline around yellow circles in the graphs (for example Figure 4), as these are difficult to see clearly in print.
**RESPONSE:** As suggested, individual data points have been amended to gray in Figures 2, 3, 4 and 6 to improve visibility against both the yellow and blue bars.

**COMMENT:** Figure 1B and similar histograms would benefit from error bars.
**RESPONSE:** We note that the histograms represent all fibers in the affected or contralateral limb across subjects at each timepoint and, thus, are not performed at the subject but rather the cohort-level, precluding the inclusion of measures of variability. We have modified the figure captions to better reflect this.

**COMMENT:** The y-axis on histograms should be labeled.
**RESPONSE:** The y-axis is labeled on the leftmost histogram panel and given that all histograms share an identical axis scale and unit, a single y-axis label was used to reduce clutter with the intent of improving readability and interpretation. This convention is consistent with standard practice for multi-panel histogram figures in the muscle physiology literature.

**COMMENT:** Histograms would be more interpretable if shown as the percentage of fibres in each category, rather than absolute counts, since fibre loss (if it had happened) would otherwise reduce counts across bins by definition.
**RESPONSE:** We considered this suggestion; however, given that fibre numbers are relatively similar across individuals and cohorts, we believe the data are more readily interpretable in the raw counts format. Additionally, raw count data also give the reader insight into the absolute differences in fibre count between Type IIa, IIx, and IIb fibres in Figures 2 and 3.

**COMMENT:** The statement on lines 495-497 would also be better aligned with field standards if such differences were expressed in relative rather than absolute terms.
**RESPONSE:** The relative change for both overall and fibre-type specific CSA are presented as LSI values ((affected CSA/contralateral CSA) *100) in addition to absolute values (mean bar) in Figures 1, 2, and 3, aligning with field standard. We have also expanded the explanation concerning the statistical comparisons concerning relative vs. absolute differences in the magnitude of CSA reductions across fibre types (lines 587-599).

**COMMENT:** I do not understand why the morphological analyses are separated by sex, whereas fibre-type composition is presented with males and females combined. If sex-specific differences are considered important for one outcome, it seems equally plausible that they could matter for the other. This should either be justified or made more consistent.
**RESPONSE:** This inconsistency reflected the underlying statistical results, although we appreciate that the rationale for this distinction was not clearly communicated in the original submission. To improve clarity, we have substantially revised the statistical and Results sections to better explain the analytical framework and rationale underlying each dataset presentation. Briefly, size-related measurements of overall and fibre-type CSA were analysed sex-stratified because the omnibus LMM revealed significant Limb x Sex ($p = 0.003$) and Limb x Sex x Timepoint ($p = 0.008$) interactions, indicating that the magnitude and time course of the contralateral-vs-injured deficit differed between males and females. In contrast, fibre-type distribution showed no significant Sex, Limb x Sex, Sex x FiberType, or higher-order Sex interactions in the omnibus LMM (all $p > 0.05$); we therefore pooled across sex when contrasting L vs R proportions to maximize power. We have amended the relevant section to explain this rationale (lines 629-637).

**COMMENT:** I have some concern about how "mis-shaped" fibres may have been handled. Fibre shape has recently been shown to be biologically meaningful (PMID: 38123165), and exclusion based on fixed thresholds may therefore bias the analysis if certain conditions disproportionately affect shape. I am not saying this necessarily occurred here, but the manuscript should more clearly explain what was excluded and why.
**RESPONSE:** We agree this is an important consideration. Exclusion rates based on CSA and circularity thresholds were examined across groups and timepoints, with no systematic difference in exclusion frequency was observed between the ACL-injured and contralateral limbs. This suggests that the injury condition did not disproportionately affect shape-based exclusions in a way that biases comparisons between limbs. We would also note that given the scale of the dataset, with thousands of fibres analyzed per section, even modest shifts

in the proportion of excluded fibres would be unlikely to meaningfully alter the group-level estimates of CSA or fibre number. We have added clarification of this point to the Methods on lines 264-267.

**COMMENT:** Relatedly, lines 240-241 require clarification: "Fibres not meeting these dominance criteria were flagged as ambiguous and subject to manual review by a trained observer." Please explain exactly what this means. Hybrid fibres will almost certainly be present, particularly in the treated condition. How were these handled? Were they assigned to the dominant signal, or treated separately? This needs to be transparent.
**RESPONSE:** Fibres that did not meet the predefined dominance criteria were classified as “Ambiguous,” reflecting the inability to confidently assign a fibre type based on fluorescence intensity alone. Across the full dataset (n = 617,353 fibres), ambiguous fibres accounted for 13,173 fibres (~2.1%), indicating that the automated classification approach successfully resolved the vast majority (~97.9%) of fibres without the need for manual reassignment. With respect to these ambiguous fibres, the current approach did not permit confident resolution of these subtypes. These fibres were conservatively retained as “Ambiguous” and not put forward for analysis rather than forced into a specific fibre-type classification. We have clarified in lines 276-287.

With respect to hybrid fibres, while hybrid fibres are expected to occur, the present immunolabeling approach is optimized for detection of Type I, IIa, IIb MyHC isoforms and does not include a specific marker for Type IIx. As a result, hybrid fibres with potential IIa/IIx or IIx/IIb co-expression cannot be reliably distinguished from fibres with low overall signal intensity or true Type IIx fibres. With respect to hybrid Type I/IIa fibres, we observed sparse Type I fibres in this dataset (~1%), reducing the likelihood of resolving biologically meaningful Type I/IIa hybrid populations. With respect to potential Type IIa/IIb hybrids, we would expect these fibres to necessarily pass through regions of low or absent staining in the unlabeled IIx dimension, making them difficult to distinguish from background or low-intensity staining.

**COMMENT:** As per journal policy, supplemental material and appendices are generally not allowed, and in any case some of these items seem too central to remain outside the main paper. The body composition data could likely be incorporated as a table, and A1 (whole-muscle IF example) seems important for interpretation of the main findings. I would strongly encourage the authors to move essential items into the main manuscript.
**RESPONSE:** We agree that these data are important enough to include in the main manuscript. We have amended the results section to include the body composition and VL wet muscle weight data, with the other quadriceps muscles reported in the appendix. Figure A1 has also been moved into the main manuscript as a representative methods figure to allow readers to evaluate the imaging approach and fibre classification procedure directly.

**COMMENT:** Lines 70-71: the sentence implying that reductions in fibre CSA reflect activation of canonical atrophy pathways is too narrow. Reduced protein synthesis is also a plausible and, in many disuse settings, central explanation (PMID: 23800384).
**RESPONSE:** The statement has been amended to include reduced protein synthesis as a plausible explanation for reductions in CSA.

**COMMENT:** Lines 71-72: the statement about fibre loss being associated with denervation and impaired reinnervation should be rephrased, as these processes may also activate atrophy pathways. The contrast as written is too sharp (PMID: 28546288).
**RESPONSE:** The statement has been revised to clarify that fibre loss commonly originates from denervation-like events, which may also activate downstream atrophic pathways.

**COMMENT:** Line 640: I think the manuscript underplays how different rodent and human fibre-type distributions are. You acknowledge this later, but the issue is substantial and should be reflected more clearly here as well.
**RESPONSE:** An additional statement has been added (lines 625-628) to contextualize the differences in fibre-type distribution between rodents and humans.

**COMMENT:** Lines 766-768: the discussion of fibre-type transitions should be toned down, particularly given how debated this remains in humans.
**RESPONSE:** The language around fibre-type transitions has been tempered at lines 853–855 to reflect ongoing debate in the human literature.

**COMMENT:** Line 198: I assume "lif" should be "tif".
**RESPONSE:** Thank you for catching this, the typo has been corrected.

**COMMENT:** The overall conclusion that no change occurred in CMAP appears reasonable based on the presented data. However, the male T3 group seems to show a substantially larger SD than the other groups, which raises some concern about either variance assumptions or technical consistency. I think this deserves checking and possibly commenting on.
**RESPONSE:** The larger SD in the male T3 group reflects biological variability rather than a technical artifact, as we confirmed supramaximal stimulation for all recordings. As CMAP was not a primary outcome, we did not comment on this in the results but have added a brief note acknowledging the elevated variance in this cohort in the results section (line 707-708).

Re: JP-RP-2026-291436R1 "**Structural Determinants of Quadriceps Atrophy Following ACL Injury: Evidence for Fibre Atrophy Without Fibre Loss or Overt Peripheral Denervation**" by Luke Stoneback, Brianna Buchanan, Hadi W Hussein, Dania Ali, Sairub Naaz, Keegan Gustafson, Leah Vaikutis, Lucas Damouni, Peter CD Macpherson, and Lindsey K Lepley

Dear Dr Lepley,

Thank you for submitting your manuscript to The Journal of Physiology. It has been assessed by a Reviewing Editor and by 2 expert referees and we are pleased to tell you that it is potentially acceptable for publication following satisfactory major revision.

Please advise your co-authors of this decision as soon as possible.

The referee reports are copied at the end of this email.

Please address all the points raised and incorporate all requested revisions or explain in your Response to Referees why a change has not been made. We hope you will find the comments helpful and that you will be able to return your revised manuscript within 2 months. If your article is NOT for a Special Issue, you may have 9 months to revise. If you require an extension, please contact journal staff: jp@physoc.org. Please note that this letter does not constitute a guarantee for acceptance of your revised manuscript.

Your revised manuscript should be submitted online using the link in your Author Tasks: Link Not Available. This link is accessible via your account as Corresponding Author; it is not available to your co-authors. If this presents a problem, please contact journal staff (jp@physoc.org). Image files from the previous version are retained on the system. Please ensure you replace or remove any files that are being revised.

If you do not wish to submit a revised version of your manuscript, you must inform our journal staff (jp@physoc.org) or reply to this email to request withdrawal. Please note that a manuscript must be formally withdrawn from the peer review process at one journal before it may be submitted to another journal.

TRANSPARENT PEER REVIEW POLICY: To improve the transparency of its peer review process, The Journal of Physiology publishes online as supporting information the peer review history of all articles accepted for publication. Readers will have access to decision letters, including Editors' comments and referee reports, for each version of the manuscript, as well as any author responses to peer review comments. Referees can decide whether or not they wish to be named on the peer review history document.

ABSTRACT FIGURES: Authors are expected to use The Journal's premium BioRender account to create/redraw their Abstract Figures. Information on how to access this account is here: https://physoc.onlinelibrary.wiley.com/journal/14697793/biorender-access.

This will enable Authors to create and download high-resolution figures. If authors have used the free BioRender service, they can use the instructions provided in the link above to download a high-resolution version suitable for publication.

The link provided should only be used for the purposes of this submission. Authors will be charged for figures created on this account if they are not related to this manuscript submission.

LANGUAGE EDITING AND SUPPORT FOR PUBLICATION: If you would like help with English language editing, or other article preparation support, Wiley Editing Services offers expert help, including English Language Editing, as well as translation, manuscript formatting, and figure formatting at www.wileyauthors.com/eeo/preparation. You can also find resources for Preparing Your Article for general guidance about writing and preparing your manuscript at www.wileyauthors.com/eeo/prepresources.

RIGOUR AND REPRODUCIBILITY: We recommend authors consult The Journal's Rigour and Reproducibility requirements to ensure that their manuscript is suitable for publication: https://physoc.onlinelibrary.wiley.com/pb-assets/hub-assets/physoc/documents/TJP-Rigour-and-Reproducibility-Requirements.pdf

REVISION CHECKLIST:

Check that your Methods section conforms to journal policy: https://jp.msubmit.net/cgi-bin/main.plex?form_type=display_requirements#methods.

Check that data presented conforms to the statistics policy: https://jp.msubmit.net/cgi-bin/main.plex?form_type=display_requirements#statistics.

Upload a full Response to Referees file. To create your 'Response to Referees': copy all the reports, including any comments from the Senior and Reviewing Editors, into a Microsoft Word, or similar, file and respond to each point, using font or background colour to distinguish comments and responses and upload as the required file type.

Please upload two versions of your manuscript text: one with all relevant changes highlighted and one clean version with no changes tracked. The manuscript file should include all tables and figure legends, but each figure/graph should be uploaded as separate, high-resolution files.

You may also upload:

- 'Potential Cover Art' for consideration as the issue's cover image
- Appropriate Supporting Information (Video, audio or data set: see https://jp.msubmit.net/cgi-bin/main.plex?form_type=display_requirements#supp).

We look forward to receiving your revised submission.

If you have any queries, please reply to this email and we will be pleased to advise.

Yours sincerely,

Paul Greenhaff
Senior Editor
The Journal of Physiology

----------------

REQUIRED ITEMS

- The Journal of Physiology funds authors of provisionally accepted papers to use the premium BioRender site to create high resolution schematic figures. Follow this link and enter your details and the manuscript number to create and download figures. Upload these as the figure files for your revised submission. If you choose not to take up this offer, we require figures to be of similar quality and resolution. If you are opting out of this service to authors, state this in the Comments section on the Detailed Information page of the submission form. The link provided should only be used for the purposes of this submission. Authors will be charged for figures created on this premium BioRender account if they are not related to this manuscript submission.

- Please include your Abstract Figure legend, which should not exceed 150 words in length, in the main manuscript file.

----------------

EDITOR COMMENTS

Please also see 'Required Items' above.

Reviewing Editor: Comments for Authors to ensure the paper complies with the Statistics Policy (Required):
See below

Comments to the Author (Required):
Thank you for the response. As you will see a number of legitimate concerns from reviewer #2 remain, which must be fully addressed before publication can be considered. I am in agreement that multiple aspects of the statistical description, outcome, and interpretation lack clarity. Please also carefully review the way in which the quantity of NMJs analysed is reported.

As an addition to those comments from the reviewer, please consider the following:

In your response you refer specifically to Figure 5 which shows RMS EMG.

Here there was no Sex x Timepoint interaction, indicating you could not confidently state males and females progressed differently. There was a main effect of group, indicating males and females differed when collapsed across all timepoints. There was a main effect of time, indicating differences across timepoints when groups were collapsed.

The justification for exploring sexes separately is not supported here, particularly in such a small sample size.

Viewing separately in this context reveals nothing of sex differences.

From the section below, please clarify the meaning of post-hoc contrasts. Are they derived from estimated marginal means? In this context, please also clarify the meaning of 'this effect was driven by', as it is not clear what effect this applies to. This may aid understanding of the interpretation.

646 - "Post-hoc contrasts indicated that this effect was driven by an acute reduction in VL EMG activity at 6 hours post-injury. In females, peak RMS EMG was reduced by 40.8% relative to T0 (p = 0.0039), whereas no other timepoints differed from baseline. In males, no timepoint significantly differed from T0, although a similar magnitude reduction was observed at 6 hours (-33.9%, p = 0.1314; Figure 5B)."

The use of 4 way interactions on this sample size should also be interpreted with caution ("Limb x Fibre Type x Sex x Timepoint as fixed effects and animal as a random intercept"), and it would be prudent to highlight this as a limitation. A more simple and informative approach would be to report the marginal and conditional R2 of each model. This output is readily available in the R packages listed in the methods.

The above is used as an example but similar issues are present with other measures (see reviewer comments). I must emphasise the importance of clarity on this issue as it influences much of the discussion.

Senior Editor:

Comments to the Author:
Thank you for the revised manuscript. Both reviewers are of the opinion that the manuscript has been improved and believe the research would be of interest to the readership of the Journal of Physiology. However, Reviewer 2 and the Reviewing Editor have raised several major concerns that impact on the interpretation of the data and currently preclude the manuscript from being acceptable for publication. Hopefully the authors can accept these concerns in the collegiate manner in which they have been presented and are open to revising the manuscript accordingly.

As stipulated in the Instructions to authors for animal experiments (https://physoc.onlinelibrary.wiley.com/hub/animal-experiments), all authors must include in the Methods section a statement that the investigators understand the animal ethical principles under which the journal operates, as laid out by Grundy (2015), O'Halloran (2024) and O'Halloran (2024). This could be placed in the section of the Methods titled "Ethical approval".

-----------------

REFEREE COMMENTS

Referee #1:

rs have addressed my concerns. Thank you for the thorough and thoughtful revision.

Referee #2:

The authors have made a solid revision and addressed many of my concerns. However, a few outstanding issues still require attention.

COMMENT: Related to this, if a linear mixed model shows no significant interaction, then post hoc testing aimed at identifying differential changes between groups over time is not justified. This appears relevant for several outcomes, including Figure 5, and should be reconsidered.

RESPONSE: We completely agree with the general principle regarding post-hoc testing following non significant omnibus tests and can confirm that post-hoc testing was only performed following significant main effects or interactions. We note that as it pertains to Figure 5, main effects for timepoint were found for both stance duration and peak RMS EMG (lines 569-579), thus post-hoc testing was appropriate.

NEW COMMENT: I think the revised statistical section is a clear improvement. However, my concern is still that some post hoc comparisons may not be justified. In a two-factor design, if the interaction is not significant, post hoc comparisons aimed at identifying differential effects between factor levels should generally be avoided (or interpreted with utmost caution). In

some cases, a significant main effect may be sufficient to conclude that an effect occurred, independent of the other factor. For example, in Fig. 4A, there appear to be main effects of fiber type and limb, but possibly no fiber type × limb interaction. If so, within-fiber-type post hoc comparisons between limbs may not be well supported. Please reconsider the post hoc testing strategy throughout the manuscript and ensure that the comparisons shown directly follow from the relevant model outputs.

COMMENT: Also, for the NMJ dataset, the biologically relevant unit is the number of NMJs analyzed per animal, not simply the total number of NMJs analyzed. Please report this more clearly.

RESPONSE: Agreed. The number of NMJs analyzed per muscle (on average) for each cohort is now reported in lines 719-721.

NEW COMMENT: I think this refers to line 688 in the version with changes marked. Just to be clear, did you analyze approximately 1000 NMJs per muscle, with two muscles per animal and n = 6 animals per group, giving approximately 12,000 NMJs in total? Please confirm or correct this interpretation. I also suggest reporting the NMJ counts as whole numbers rather than decimals.

COMMENT: I missed a clear rationale for focusing specifically on the VL muscle, and for restricting the analysis to that muscle alone. This should be explained.

RESPONSE: The following rationale has now been added to the manuscript. "The VL was selected to facilitate comparison with prior preclinical work in this ACL injury model (Davi et al., 2022; Hunt et al., 2022; Noh et al., 2024) and to enhance translational relevance to human biopsy-based studies (Refs), where the VL is the most commonly sampled quadriceps muscle." Lines 129-132.

NEW COMMENT: This added rationale is helpful. However, the VL does not necessarily have the same functional role or fiber-type composition in rodents as in humans, and muscle-specific responses to ACL injury may differ across quadriceps and other hindlimb muscles. I therefore still think the manuscript should acknowledge, as a separate limitation, that the analysis was restricted to one muscle and that different muscles may adapt differently. This would complement the existing limitations related to fiber-type composition.

---

END OF COMMENTS

**SENIOR EDITOR**

**COMMENT:** Thank you for the revised manuscript. Both reviewers are of the opinion that the manuscript has been improved and believe the research would be of interest to the readership of the Journal of Physiology. However, Reviewer 2 and the Reviewing Editor have raised several major concerns that impact on the interpretation of the data and currently preclude the manuscript from being acceptable for publication. Hopefully the authors can accept these concerns in the collegiate manner in which they have been presented and are open to revising the manuscript accordingly. As stipulated in the Instructions to authors for animal experiments

(https://physoc.onlinelibrary.wiley.com/hub/animal-experiments), all authors must include in the Methods section a statement that the investigators understand the animal ethical principles under which the journal operates, as laid out by Grundy (2015), O'Halloran (2024) and O'Halloran (2024). This could be placed in the section of the Methods titled "Ethical approval".

**RESPONSE:** We thank the Editor and both reviewers for their continued constructive feedback. In response to the concerns raised, we have revised our post hoc testing strategy throughout the manuscript to ensure comparisons follow directly from significant model terms, added marginal and conditional $R^2$ for all linear mixed models, added a limitation addressing the use of higher-order interactions given our sample size, and clarified NMJ counts and the rationale/limitation for restricting analysis to the VL. We have also added the required statement of compliance with the journal's animal ethical principles (Grundy, 2015; O'Halloran, 2024) to the "Ethical approval" subsection of the Methods.

**REVIEWING EDITORS**

**COMMENT:** Thank you for the response. As you will see a number of legitimate concerns from reviewer #2 remain, which must be fully addressed before publication can be considered. I am in agreement that multiple aspects of the statistical description, outcome, and interpretation lack clarity. Please also carefully review the way in which the quantity of NMJs analysed is reported.
**RESPONSE:** We thank the Editor for this feedback. To address these concerns, we revised the statistical description, outcome reporting, and interpretation throughout the manuscript. Specifically, we ensured that post hoc analyses were performed only for the appropriate main effects and interaction terms, added $R^2$ reporting for all mixed models, and revised the NMJ quantification (lines 780-784) to clearly state that approximately 1,000 NMJs were analyzed per muscle, across two muscles per animal and n = 6 animals per sex (12 animals total), for approximately 24,000 NMJs evaluated overall. We also now report these values as whole numbers. We have gone through each of Reviewer 2's remaining points individually below and describe the corresponding revisions.

**COMMENT:** As an addition to those comments from the reviewer, please consider the following:
In your response you refer specifically to Figure 5 which shows RMS EMG. Here there was no Sex x Timepoint interaction, indicating you could not confidently state males and females progressed differently. There was a main effect of group, indicating males and females differed when collapsed across all timepoints. There was a main effect of time, indicating differences across timepoints when groups were collapsed. The justification for exploring sexes separately is not supported here, particularly in such a small sample size.
Viewing separately in this context reveals nothing of sex differences.
**RESPONSE:** We agree and have revised the results and discussion pertaining to the stance duration and EMG data accordingly. Specific to Figure 5 (stance duration and peak RMS EMG): Neither outcome showed a significant Sex x Timepoint interaction (stance duration: $F(4,147.59) = 0.81$, $p = 0.5201$; EMG: $F(4,142.73) = 0.25$, $p = 0.9081$). We have therefore replaced the sex-stratified post hoc contrasts with contrasts pooled across sex for both outcomes, and revised Figure 5 to display pooled estimated marginal means rather than sex-stratified trajectories.

**COMMENT:** From the section below, please clarify the meaning of post-hoc contrasts. Are they derived from estimated marginal means? In this context, please also clarify the meaning of 'this effect was driven by', as it is not clear what effect this applies to. This may aid understanding of the interpretation.

> 646 - "Post-hoc contrasts indicated that this effect was driven by an acute reduction in VL EMG activity at 6 hours post-injury. In females, peak RMS EMG was reduced by 40.8% relative to T0 ($p = 0.0039$), whereas no other timepoints differed from baseline. In males, no timepoint significantly differed from T0, although a similar magnitude reduction was observed at 6 hours (-33.9%, $p = 0.1314$; Figure 5B)."

**RESPONSE:** We have also clarified in the text that post hoc contrasts are derived from estimated marginal means (emmeans) applied to the fitted linear mixed models, and revised language such as 'this effect was driven by' to explicitly name the Timepoint main effect being decomposed.

**COMMENT:** The use of 4-way interactions on this sample size should also be interpreted with caution ("Limb x Fibre Type x Sex x Timepoint as fixed effects and animal as a random intercept"), and it would be prudent to highlight this as a limitation. A more simple and informative approach would be to report the marginal and conditional R2 of each model. This output is readily available in the R packages listed in the methods.
The above is used as an example, but similar issues are present with other measures (see reviewer comments). I must emphasise the importance of clarity on this issue as it influences much of the discussion.
**RESPONSE:** We agree and have added marginal and conditional $R^2$ for each linear mixed model in the manuscript, computed using the performance package as suggested. We have also added a limitation noting that higher-order interaction terms (e.g., Limb x Fibre Type x Sex x Timepoint) are likely underpowered given our sample size.

**REFEREE #1**

**COMMENT:** rs have addressed my concerns. Thank you for the thorough and thoughtful revision.
**RESPONSE:** We thank the reviewer for their time spent reviewing the manuscript and feedback throughout.

**REFEREE #2**

**COMMENT:** I think the revised statistical section is a clear improvement. However, my concern is still that some post hoc comparisons may not be justified. In a two-factor design, if the interaction is not significant, post hoc comparisons aimed at identifying differential effects between factor levels should generally be avoided (or interpreted with utmost caution). In some cases, a significant main effect may be sufficient to conclude that an effect occurred, independent of the other factor. For example, in Fig. 4A, there appear to be main effects of fiber type and limb, but possibly no fiber type × limb interaction. If so, within-fiber-type post hoc comparisons between limbs may not be well supported. Please reconsider the post hoc testing strategy throughout the manuscript and ensure that the comparisons shown directly follow from the relevant model outputs.
**RESPONSE:** We agree with this principle and have revised our post hoc testing strategy throughout the manuscript so that comparisons are only decomposed to the level of detail supported by a significant interaction term.

Figure 5 (stance duration and peak RMS EMG): Neither outcome showed a significant Sex x Timepoint interaction (stance duration: $F(4,147.59) = 0.81$, $p = 0.5201$; EMG: $F(4,142.73) = 0.25$, $p = 0.9081$). We have therefore replaced the sex-stratified post hoc contrasts with contrasts pooled across sex for both outcomes, and revised Figure 5 to display pooled estimated marginal means rather than sex-stratified trajectories. For stance duration, pooled contrasts showed a significant reduction at 6 hours post-injury relative to baseline ($\Delta = -0.033$ s, $p = 0.0013$), with no significant differences at 3, 7, or 14 days. For EMG, pooled contrasts showed a significant reduction at 6 hours (-37.5%, $p = 0.0013$), with no significant differences at later timepoints. We have also clarified in the text that post hoc contrasts are derived from estimated marginal means (emmeans) applied to the fitted linear mixed models, and revised language such as 'this effect was driven by' to explicitly name the effect being decomposed.

Figure 4A / Table 3 (fibre-type composition): We reviewed this analysis against the same principle. None of the Sex x Limb interaction terms were significant (Limb x Sex $p = 0.936$; Limb x FibreType x Sex $p = 0.090$; Limb x Sex x Timepoint $p = 0.974$; Limb x FibreType x Sex x Timepoint $p = 0.268$), justifying pooling across sex for the affected-versus-contralateral contrasts. The Limb x Fibre Type interaction was significant ($F(2,180) = 4.49$, $p = 0.012$), justifying decomposition of the limb contrast by fibre type. However, the Limb x Fibre Type x Timepoint interaction was only a trend ($F(4,180) = 2.30$, $p = 0.061$). As such, we have revised the text to state explicitly that the timepoint-specific contrasts characterize this significant two-way effect rather than confirm a statistically distinct temporal trajectory and have softened related interpretive language in the Discussion accordingly (e.g., 'trends toward' rather than assertions of a confirmed progressive shift).

**COMMENT:** I think this refers to line 688 in the version with changes marked. Just to be clear, did you analyze approximately 1000 NMJs per muscle, with two muscles per animal and n = 6 animals per group, giving approximately 12,000 NMJs in total? Please confirm or correct this interpretation. I also suggest reporting the NMJ counts as whole numbers rather than decimals.
**RESPONSE:** We thank the reviewer for flagging this ambiguity. To clarify: we analyzed approximately 1000 NMJs per muscle, with two muscles (contralateral and affected VL) evaluated per animal, and n = 6 animals per sex (12 animals total, not 6). This gives approximately 24,000 NMJs evaluated in total, rather than the ~12,000 suggested by the reviewer's interpretation. We have revised the text at lines 780-784 to state this explicitly and to report NMJ counts as whole numbers rather than decimals throughout.

**COMMENT:** This added rationale is helpful. However, the VL does not necessarily have the same functional role or fiber-type composition in rodents as in humans, and muscle-specific responses to ACL injury may differ across quadriceps and other hindlimb muscles. I therefore still think the manuscript should acknowledge, as a separate limitation, that the analysis was restricted to one muscle and that different muscles may adapt differently. This would complement the existing limitations related to fiber-type composition.
**RESPONSE:** We agree and have added a separate limitation acknowledging that our analysis was restricted to the VL muscle. While the VL was selected for its translational relevance to human biopsy studies and comparability with prior work in this model, we recognize that responses to ACL injury may vary across the quadriceps. This has been added as a distinct limitation in addition to the pre-existing limitation discussing differences in fibre-type composition between humans and rodents (lines 980-984).

**2nd Revision - Editorial Decision** **28-Jul-2026**

Dear Dr Lepley,

Re: JP-RP-2026-291436R2 "**Structural Determinants of Quadriceps Atrophy Following ACL Injury: Evidence for Fibre Atrophy Without Fibre Loss or Overt Peripheral Denervation**" by Luke Stoneback, Brianna Buchanan, Hadi W Hussein, Dania Ali, Sairub Naaz, Keegan Gustafson, Leah Vaikutis, Lucas Damouni, Peter CD Macpherson, and Lindsey K Lepley

We are pleased to tell you that your paper has been accepted for publication in The Journal of Physiology.

The last Word (or similar) version of the manuscript provided will be used by the Production Editor to prepare your proof. When this is ready you will receive an email containing a link to Wiley's Online Proofing System. The proof should be thoroughly checked and corrected as promptly as possible.

Authors should note that it is too late at this point to offer corrections prior to proofreading. Major corrections at proof stage, such as changes to figures, will be referred to the Editors for approval before they can be incorporated. Only minor changes, such as to style and consistency, should be made at proof stage. Changes that need to be made after proof stage will usually require a formal correction notice.

All queries at proof stage should be sent to: TJP@wiley.com.

Following copyediting and typesetting the accepted version will be published online.

Yours sincerely,

Paul Greenhaff
Senior Editor
The Journal of Physiology

IMPORTANT POINTS TO NOTE FOLLOWING ACCEPTANCE OF YOUR PAPER:

• IMPORTANT NOTICE ABOUT OPEN ACCESS: To assist authors whose funding agencies mandate immediate public access to published research findings, The Journal of Physiology allows authors to pay an Open Access (OA) fee to have their papers made freely available immediately on publication.

The Corresponding Author will receive an email from Wiley with details on how to register or log in to Wiley Authors where you will be able to place an order.

• You can check if your funder or institution has a Wiley Open Access Account here:
https://authors.wiley.com/author-resources/Journal-Authors/open-access/author-compliance-tool.html

MEMBER DISCOUNT: Corresponding authors who are Members of the Physiological Society are eligible for a 20% discount on the cost of the Article Processing Charge (APC). To claim this discount, the Corresponding Author will need to log in to the Physiological Society Membership portal and retrieve the discount code (https://www.physoc.org/journals-and-media/journals/journal-discount-for-members/). This code should then be quoted in the billing section of Wiley Authors when selecting the open access option.

• TRANSPARENT PEER REVIEW POLICY: To improve the transparency of its peer review process, The Journal of Physiology publishes online as supporting information the peer review history of all articles accepted for publication. Readers will have access to decision letters, including Editors' comments and referee reports, for each version of the manuscript as well as any author responses to peer review comments. Referees can decide whether or not they wish to be named on the peer review history document.

• You can help your research get the attention it deserves! Check out Wiley's free Promotion Guide for best-practice recommendations for promoting your work at: www.wileyauthors.com/eeo/guide. You can learn more about Wiley Editing Services which offers professional video, design, and writing services to create shareable video abstracts, infographics, conference posters, lay summaries, and research news stories for your research at: www.wileyauthors.com/eeo/promotion.

• If you would like to receive our 'Research Roundup', a monthly newsletter highlighting the cutting-edge research published in The Physiological Society's family of journals (The Journal of Physiology, Experimental Physiology, Physiological Reports, The Journal of Nutritional Physiology and The Journal of Precision Medicine: Health and Disease), please click this link, fill in your name and email address and select 'Research Roundup':
https://www.physoc.org/journals-and-media/membernews

----------------

EDITOR COMMENTS

Reviewing Editor:

Comments to the Author (Required):
Thank you for the additional clarification.

Senior Editor:

Comments to the Author:
Thank you for the revised manuscript and rebuttal document, which have been considered by the same reviewing editor and reviewers that considered the original manuscript submission. I am happy to convey that all believed the authors have addressed the previous review comments adequately and that the revised manuscript has been improved. The manuscript is now deemed acceptable for publication. Congratulations and thank you for supporting The Journal of Physiology.

-----------------

REFEREE COMMENTS

Referee #1:

No additional concerns.

Referee #2:

Nothing further to add
